# Childhood Adversities and Psychosis Across Populations: Insights From the 6-Country EU-GEI Study

**DOI:** 10.1093/schbul/sbag030

**Published:** 2026-05-04

**Authors:** Craig Morgan, Charlotte Gayer-Anderson, Lucia Sideli, Hannah E Jongsma, Eva Velthorst, Ilaria Tarricone, Laura Ferraro, Daniele La Barbera, Caterina La Cascia, Antonio Lasalvia, Sarah Tosato, Domenico Berardi, Celso Arango, Manuel Arrojo, Miguel Bernardo, Julio Bobes, Julio Sanjuán, Jose Luis Santos, Paulo Menezes Rossi, Cristina Marta Del-Ben, Pierre-Michel Llorca, Andrea Tortelli, Andre Szoke, Lieuwe de Haan, Jean-Paul Selten, Jim Van Os, Bart P Rutten, Marta Di Forti, Peter B Jones, James B Kirkbride, Robin M Murray

**Affiliations:** Institute of Psychiatry, Psychology & Neuroscience, King’s College London, London, SE5 8AB, United Kingdom; ESRC Centre for Society and Mental Health, King’s College London, London, SE5 8AB, United Kingdom; Institute of Psychiatry, Psychology & Neuroscience, King’s College London, London, SE5 8AB, United Kingdom; ESRC Centre for Society and Mental Health, King’s College London, London, SE5 8AB, United Kingdom; Department of Human Science, LUMSA University, 00193 Rome, Italy; Department of Psychiatry, University of Cambridge, Cambridge, CB2 2QQ, United Kingdom; Departmentof Community Mental Health, GGZ Noord-Holland-Noord, 1850 BA, Heerhugowaard, the Netherlands; Department of Medical and Surgical Sciences, Alma Mater Studiorum, Bologna University, 40126 Bologna, Italy; Department of Biomedicine, Neuroscience, and Advanced Diagnostic, University of Palermo, 90133 Palermo, Italy; Department of Biomedicine, Neuroscience, and Advanced Diagnostic, University of Palermo, 90133 Palermo, Italy; Department of Biomedicine, Neuroscience, and Advanced Diagnostic, University of Palermo, 90133 Palermo, Italy; Section of Psychiatry, Department of Neuroscience, Biomedicine and Movement, University of Verona, 37134 Verona, Italy; Section of Psychiatry, Department of Neuroscience, Biomedicine and Movement, University of Verona, 37134 Verona, Italy; Department of Biomedical and NeuroMotor Sciences, Psychiatry Unit, Alma Mater Studiorum Università di Bologna, 40126 Bologna, Italy; Institute of Psychiatry and Mental Health, Department of Child and Adolescent Psychiatry, Hospital General Universitario Gregorio Marañón, School of Medicine, Universidad Complutense, ISGM, CIBERSAM, 28007 Madrid, Spain; Department of Psychiatry, Psychiatric Genetic Group, Instituto de Investigación Sanitaria de Santiago de Compostela, Complejo Hospitalario Universitario de Santiago de Compostela, 15706 Santiago, Spain; Barcelona Clinic Schizophrenia Unit, Hospital Clinic de Barcelona, Institul d’Investigacions Biomèdiques August Pi i Sunyer (IDIBAPS) Barcelona, 08017 Barcelona, Spain; Department of Medicine, Psychiatry Area, School of Medicine, Universidad de Oviedo, ISPA, INEUROPA, Centro de Investigación Biomédica en Red de Salud Mental (CIBERSAM), 33011 Oviedo, Spain; Department of Psychiatry, School of Medicine, Universidad de Valencia, Centro de Investigación Biomédica en Red de Salud Mental, 46010 Valencia, Spain; Department of Psychiatry, Hospital “Virgen de la Luz”, 16003 Cuenca, Spain; Department of Preventive Medicine, Faculdade de Medicina, Universidade de São Paulo, São Paulo, 01246-903, Brazil; Division of Psychiatry, Department of Neuroscience and Behaviour, Ribeirão Preto Medical School, Universidade de São Paulo, Ribeirão Preto - SP, 14051-140, Brazil; Université Clermont Auvergne, 63000 Clermont-Ferrand, France; Establissement Public de Santé, Maison Blanche, 75020 Paris, France; Univ Paris Est Creteil, INSERM, IMRB, AP-HP, Hôpitaux Universitaires « H. Mondor », DMU IMPACT, Fondation Fondamental, F-94010 Creteil, France; Departmentof Community Mental Health, GGZ Noord-Holland-Noord, 1850 BA, Heerhugowaard, the Netherlands; Institute for Mental Health, GGZ Rivierduinen, 2333 ZZ, Leiden, The Netherlands; Department of Psychiatry and Neuropsychology, School for Mental Health and Neuroscience, Maastricht University Medical Centre, 6229 ER, Maastricht, The Netherlands; ESRC Centre for Society and Mental Health, King’s College London, London, SE5 8AB, United Kingdom; Department of Psychiatry and Neuropsychology, School for Mental Health and Neuroscience, Maastricht University Medical Centre, 6229 ER, Maastricht, The Netherlands; Department of Psychiatry, Utrecht University Medical Centre, 3584 CX, Utrecht, The Netherlands; Department of Psychiatry and Neuropsychology, School for Mental Health and Neuroscience, Maastricht University Medical Centre, 6229 ER, Maastricht, The Netherlands; ESRC Centre for Society and Mental Health, King’s College London, London, SE5 8AB, United Kingdom; South London and Maudsley NHS Foundation Trust, London, SE5 8AZ, United Kingdom; Department of Psychiatry, University of Cambridge, Cambridge, CB2 2QQ, United Kingdom; PsyLife Group, Division of Psychiatry, University College London, London, W1T 7AD, United Kingdom; ESRC Centre for Society and Mental Health, King’s College London, London, SE5 8AB, United Kingdom

**Keywords:** clusters of adversity, threat, hostility, violence, population rates

## Abstract

**Background and Hypothesis:**

Using data from the EU-GEI Work Package 2 (EU-GEI WP2) programme, we sought to test several hypotheses related to gaps in our knowledge of associations between childhood adversities and psychosis.

**Study Design:**

EU-GEI WP2 comprises incidence and case–control studies of first-episode psychosis conducted in 17 sites in 6 countries. In each site, over 2-year periods, we identified and collected relevant data from individuals aged 18-64 with a first-episode psychosis and with no history of psychosis. Missing data were imputed. We used multi-level logistic regression to test our hypotheses.

**Study Results:**

In total, 1071 cases and 1497 controls were included. We found variations in the prevalence and the magnitude of associations between any adversity and psychosis by place (eg, odds ratios ranged from 0.4 [Cuenca, Spain] to 12.1 [Madrid, Spain]). The weighted percentages reporting adversities in control samples were associated with site incidence rates (eg, 3+ adversities: Spearman’s rho 0.56, *P* .025). We found variations in the magnitude of associations by sex (eg, effect of physical and sexual abuse stronger among women), by age of exposure, and by severity and frequency of adversities (eg, largest odds ratios for adversities involving hostility, threat, and violence).

**Conclusions:**

Variations across populations in prevalence and effects of adversities may contribute to variations in rates of psychosis. Variations in effects by sex and age of onset may point to sex-specific mechanisms and to developmentally sensitive periods. Adversities involving severe threat, hostility, and violence may have the largest effects on risk of psychosis.

## Introduction

A substantial body of research has found consistent evidence of an association between childhood adversities and psychoses, with odds ratios typically in the range of 2-4.^1–6^ In recent years, this research has deepened to examine more fine-grained aspects of these associations, generating novel insights on cumulative and synergistic effects,^7^^,^^8^ gene–adversity correlation and interaction,^9^^,^^10^ mediating biological and psychological mechanisms,^11–17^ and associations with diagnosis and specific symptoms.^18–22^

However, a recent review of 585 studies since 2010 highlighted several limitations that characterize much of the research to date.^23^ First, there is a lack of conceptual clarity. Various terms and concepts have been used to describe the experiences being studied, often interchangeably, including adversities, trauma, maltreatment, and victimization, typically with no clear justification, theoretical or otherwise, or definition. This hinders comparability and contributes to a lack of conceptual clarity and inconsistencies in the terms used to refer to similar experiences. Second, most studies have focused on a narrow range of experiences, eg, sexual and physical abuse, reflecting what has been termed a child maltreatment–centered approach.^24^ This focus means that other social adversities, such as parental death and separation, domestic violence, bullying, violence and discrimination, and poverty, have received relatively less attention. Third, there has been limited consideration of whether the effects of adversities vary by sex. This is important. There are well-documented differences between boys and girls in the type and frequency of adversities experienced (eg, sexual abuse more common among girls^25^) and some evidence that effects vary by sex (ie, stronger effects among girls/women compared with boys/men).^26^^,^^27^ Given this, analyses testing hypotheses about differences by sex should, arguably, be routine in all studies of adversity and psychosis. Fourth, other dimensions of adversity, such as timing, frequency, and severity, have not been routinely considered. This limits our understanding of complex ways in which adversity is linked to psychosis over the course of development. Variations in experience along these dimensions may partly explain why only a fraction of those exposed to adversities ever develop a psychotic disorder. It is plausible, for example, that it is frequent, severe adversities occurring at developmentally critical stages that have the most profound and enduring effects on psychological and biological processes that may underlie the emergence of psychosis. We found some evidence to support this in analyses of data from a study in London, UK, but these findings require replication and extension.^28^ Fifth, most studies have focused solely on adversities and not examined the potential protective (or resilience) effects of positive experiences and relationships, either in general or as modifiers of the effects of adversities. The challenge with examining protective factors, for example, social supports, in mitigating the effects of adversities is that data are often not refined enough. For example, the measure of support does not relate to the time period in which the adversity occurred.^29^ However, there is value in testing hypotheses concerning the general protective effects of social supports and other positive experiences, given these may promote good mental health and thereby reduce overall risk. In this study, we sought to address the gaps identified here by testing several related hypotheses (see below).

What has been studied in more depth is the relationship between childhood adversities and the phenomenology of psychosis.^18^ Broadly, this line of research suggests that childhood maltreatment is associated with more positive and affective symptoms. It is therefore plausible that variations in presenting symptoms and diagnosis, in part at least, reflect differences in exposure to the varied risks for psychosis, and, as with sex, this suggests studies should routinely examine this in analyses by stratifying by diagnosis.

At a population level, the extent, nature, co-occurrence, and meaning of childhood adversities vary by context, by social group, and over time.^30^ Adversities are socially patterned, reflecting wider structural inequities that both increase risk of exposure and limit access to material and other resources that may mitigate their effects.^31^ Here, eco-social theories of health, which situate individual and interpersonal experiences within historical and social context, are relevant.^32^ This means that the effects and impacts of adversities on risk of psychosis are likely to vary by place, group, and over time. Indeed, there is some evidence that this is the case for other risks, such as cannabis use,^33^ and this points to the importance of considering variations by place in the distribution, effects, and impacts of adversities and other risks. However, we are not aware of any studies that have compared the effects and impacts of childhood adversities on psychosis in multiple contexts simultaneously, using the same concepts and methods.

Here, we present analyses of data from the multi-site EU-GEI Work Package 2 (WP2) programme^34^ on childhood adversities that sought to replicate previous studies and, for the first time, test hypotheses related to the gaps in understanding noted above by considering a wide range of adversities (ie, challenging contexts, situations, and experiences that may be stressful or traumatic), cumulative effects, and variations by sex, diagnosis, and place, and by type, frequency, age of exposure, and severity. Specifically, we sought to test the following 5 hypotheses:

(H1) Each form of adversity will be associated with increased odds of psychotic disorder.

(H2) Odds of psychosis will increase with each additional adversity reported.

(H3) The effects of each adversity will vary by sex (ie, greater for women) and diagnosis (ie, greater for affective psychoses)

(H4) The effect of any adversity on odds of psychosis and the prevalence of any adversity will vary by site, with prevalence in controls (ie, proxy population estimate) positively correlated with incidence of psychosis.

(H5) The effects of each adversity will be greatest for more severe and frequent exposure and when first experienced at a younger age (ie, 0-11 years)

## Methods

EU-GEI WP2 is a population-based incidence and case–control study of first-episode psychosis conducted in defined catchment areas in 17 sites in 6 countries (England, Netherlands, France, Spain, Italy, Brazil) over a 5-year period (2010-2015).^34^

### Sample

In each site, we sought to identify all individuals aged 18-64 years and resident in the catchment area who presented to mental health services for a first time with a psychotic disorder (ICD-10 codes F20-29 and F30-33 [with psychotic symptoms]) during, on average, two-year study periods (ie, incident cases). All who met these inclusion criteria were invited to participate in the case–control arm of the study, and informed consent was sought. Exclusion criteria were: evidence of psychotic symptoms precipitated by an organic cause (ICD-10: F09); transient psychotic symptoms resulting from acute intoxication (F1X.5); severe learning disabilities, defined by an IQ less than 50 or diagnosis of intellectual disability (F70-F79); and insufficient fluency of the primary language in each site to complete assessments. Simultaneously, in each site, we recruited controls with no history of psychosis from the populations at risk using a mix of random and quota sampling to improve the extent to which samples reflected the age, sex, and ethnic composition of the population at risk.

### Data Collection (1) Childhood Adversities

**Figure 1:**
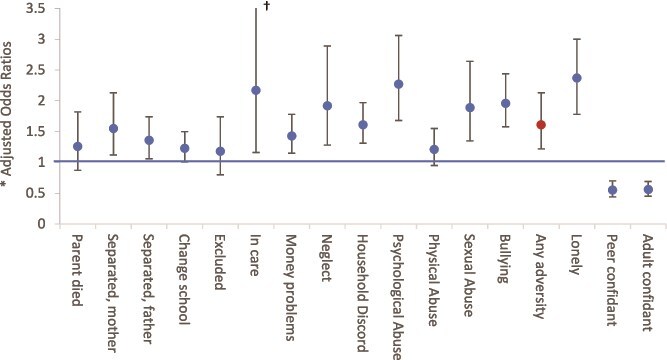
Main Effects for Each Adversity (Present vs. Absent) on Outcome (Case–Control Status) (See Supplementary Table S3 for Full Data). *Adjusted for age, sex, ethnicity, IQ, parental history of psychosis, and parental social class at birth. †95% confidence interval line truncated, upper limit: 4.06.

We collected data on 13 indicators of childhood adversities and 3 indicators of loneliness and social support (Supplementary Materials: Appendix 2; see Figure 1 for list of indicators) before age 17 years using the Childhood Experience of Care and Abuse (CECA) Questionnaire, expanded to include sections from the full CECA Interview Schedule on household discord, psychological abuse, physical abuse, sexual abuse, and bullying.^35^^,^^36^ The expanded sections probed occurrence, severity, frequency, and age at first of exposure. All ratings were made by researchers based on concrete descriptions of experiences. Severity was rated on a 4-point scale: none, some, moderate, and marked, with the exception of household discord, which included an additional point to capture domestic violence. See Appendix 3 for definitions. Of note, the highest severity ratings require the occurrence of physical violence and intense and pervasive abuse. Frequency was rated as never, rare (once or twice), occasional (more than twice, less than monthly), frequent (monthly), or very frequent (weekly) and dichotomized for analyses into frequent (monthly or more often) versus other (less than monthly). Age at exposure was defined as age at first occurrence of adversity and dichotomized for analyses into 0-11 years old (childhood) and 12-16 years old (adolescence). In CECA ratings, there was a high level of interrater reliability among researchers across sites (kappa: 0.82).^34^ For the expanded sections, we used life-course interview techniques, including anchoring by key dates, to aid recall.

### Data Collection (2) Demographic, Clinical, and Other Data

We collected data on demographic characteristics, parental social class at birth, and social circumstances using the Medical Research Council (MRC) Sociodemographic Schedule.^37^ Ethnic group was self-ascribed and coded into 7 categories to enable comparisons across sites: ie, White majority, Black, Mixed, Asian, North African, White minority, and other. Social class was defined according to the European Socio-Economic Classification^38^ system (interrater reliability: kappa 0.81).^34^ We collected symptom data using semi-structured interviews in each site; these data were used to complete the Operational Criteria Checklist for Psychotic and Affective Disorders^39^ (interrater reliability: kappa 0.70),^34^ from which we derived DSM-IV and ICD-10 diagnoses for cases. Diagnoses were grouped into schizophrenia, other non-affective psychoses, and affective psychoses. Age at onset was assessed and estimated using the Nottingham Onset Schedule. We collected information on participants’ family history of mental illness using the Family Interview for Genetic Studies.^40^ For analyses, parental history of psychosis was used as a proxy for genetic risk. We collected neurocognitive data using the WAIS-Shortened,^41^ from which we derived an IQ score.

### Ethics

All procedures were approved by the relevant local ethics committees (Supplementary Materials: Appendix 1). All participants gave written informed consent.^34^ All procedures contributing to this work comply with the ethical standards of the relevant national and institutional committees on human experimentation and with the Helsinki Declaration of 1975, as revised in 2008.

### Missing Data

To handle missing data, we used multiple imputation by chained equations.^42^^,^^43^ The imputation models included all variables in the main analyses and several auxiliary variables. Post-imputation analyses combined estimates across 25 imputed data sets using Rubin’s rule.^44^

### Analyses

With imputed data, we used multilevel logistic regression, with random intercepts at the site level to account for the hierarchical structure of the data (ie, individuals clustered within sites), to estimate odds ratios with 95% confidence intervals. In all analyses, we estimated unadjusted and adjusted odds ratios, first controlling for age, sex, and ethnicity and then for age, sex, ethnicity, IQ, parental history of psychosis, and parental social class at birth. We estimated odds ratios for each adversity separately. We did not adjust any models for other adversities to avoid issues with collinearity, given each adversity was strongly associated with others.

For H1, we estimated main effects for adversities, both overall (ie, 0 vs. 1 or more adversities; with sensitivity analyses for 0-2 vs. 3 or more adversities presented in supplementary tables) and for each adversity. For overall effects, sensitivity analyses using 0-2 vs. 3 adversities enabled us to examine the implications for our findings of using a higher cut point to create a binary variable for exposure to adversity. For H2, we created an index of childhood adversity, counting the number of adversities participants reported, and estimated odds ratios (1) for each level (with 0 adversities as the reference category) and (2) for the average increase in odds of psychosis for each additional adversity. For H3, we estimated effects for each adversity by sex, fitting interaction terms to logistic regression models, and by diagnosis (ie, controls vs. non-affective and affective psychoses separately). For H4, we fit interaction terms for any adversity (ie, 0 vs. 1 or more) × site to logistic regression models and used likelihood ratio tests to assess variation by site in the effect of any adversity on odds of psychosis. Extending this, we then examined associations between the prevalence of adversities (ie, any, 3 or more, and any at moderate or marked severity) among controls in each site, a proxy for population prevalence, and incidence rates (see Jongsma et al^45^). We first estimated unweighted and weighted prevalences of adversities among controls in each site. To weight estimates, we generated post-stratification weights to adjust for differences by age, sex, and ethnic group between our control samples and the population at risk in each site.^46^ We then estimated correlations between prevalences of adversities and incidence rates using Spearman’s rank order correlations. For H5, we estimated main effects for each level of severity, frequency, and age at first exposure (ie, 0-11 vs. 12-16 years) for the adversities we assessed in more detail.

Analyses were conducted using Stata MP version 16.0.^47^

## Results

### Sample

During the study periods, across all sites, 1130 individuals with a first-episode psychosis (41% of 2774 incident cases) and 1497 controls provided informed consent to participate in the case–control arm of the programme. We excluded cases from the Paris site (*n*, 36) where no controls were recruited and we excluded cases with an onset of psychosis before age 17 (*n*, 23), giving a final analysis sample of 1071 cases and 1497 controls. By sex, the control sample was broadly representative of the populations at risk in each site; by age, the control samples tended to be younger; and by ethnic group, there was variation by site in how representative control samples were but not in a consistent direction (Supplementary Table S1). The proportions of missing data on most variables were generally small, including each indicator of adversity (ie, less than 5% for cases and controls), with the exceptions of: any adversity for cases (8%); parental social class for cases (13%), parent history of psychosis for cases (13% and controls (11%); and IQ for cases (38%) and controls (32%) (see Supplementary Table S2a and S2b). In line with established associations, compared with controls, cases were younger and comprised more men, more from minoritized ethnic groups, more with lower levels of education, more unemployed, and more with a parent with a history of psychosis (Supplementary Table S2a and S2b). Around 49% of cases met criteria for a diagnosis of schizophrenia, 22% for other non-affective psychosis, and 28% for an affective psychosis.

### Main and Cumulative Effects (H1-2)

Overall, around 88% of cases (*n*, 869) reported 1 or more adversity vs. around 80% of controls (*n*, 1156; adj. OR 1.61, 95% CI 1.22-2.13) (Figure 1; Supplementary Table S3). There was variation in both frequency of exposure and magnitude of effect for each adversity (Figure 1; Supplementary Table S3). The most commonly reported adversities were changing school (39% of controls; 46% of cases) and household discord (38% of controls; 49% of cases) and the least common was being in care (1% of controls; 6% of cases). In fully adjusted models, the weakest associations were for exclusion from school (adj. OR 1.18, 95% CI 0.80-1.74), physical abuse (adj. OR 1.21, 95% CI 0.95-1.54), and parent died (adj. OR 1.26, 95% CI 0.87-1.82) and the strongest was for psychological abuse (adj. 2.27, 95% CI 1.68-3.06). Cases were more likely to report having been lonely (adj. OR 2.37, 95% CI 1.88-3.00) and less likely to report having close peer (adj. OR 0.55, 95% CI 0.44-0.70) or adult (adj. OR 0.56, 95% CI 0.45-0.69) confidants (Figure 1; Supplementary Table S3).

**Figure 2:**
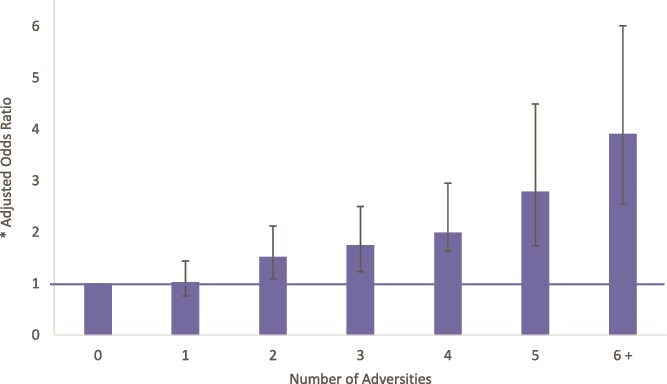
Number of Adversities and Psychotic Disorders (See Supplementary Table S5 for Full Data). Note (1) the number of adversities entered as a continuous variable: fully Adj. OR 1.25 (95% CI 1.18-1.33); for each additional adversity, odds of psychotic disorder increase by, on average, around 25%.

Among cases and controls, most forms of adversity tended to be associated with other forms of adversity (Supplementary Table S4) and we found strong evidence of a cumulative effect, such that odds of psychosis increased in linear fashion with each additional adversity reported (Figure 2; Supplementary Table S5). For example, after adjusting for putative confounders, each additional adversity was associated with, on average, a 25% increased odds of psychosis (adj. OR 1.25, 95% CI 1.18-1.33).

### By Sex, Age, and Diagnosis (H3)

By sex, for some adversities, unadjusted odds ratios were higher for men vs. women (ie, neglect, *P* for interaction .007); for others, the unadjusted odds ratio were lower for men (ie, physical abuse, *P* for interaction .016; more tentatively, sexual abuse, *P* for interaction .27); and for the remaining adversities, there were no differences. When adjusted for potential confounders, the noted differences were attenuated slightly (Supplementary Table S6). By diagnosis, there were some differences, but these did not converge to suggest a consistent pattern of associations. That is, for some adversities, the odds ratios were modestly higher for those with a non-affective psychosis vs. affective psychosis (ie, parent died, in care, physical abuse); for bullying, the odds ratio was modestly lower; and for the remaining adversities, there were no clear differences by broad diagnostic group (Supplementary Table S7).

### By Site (H4)

**Figure 3:**
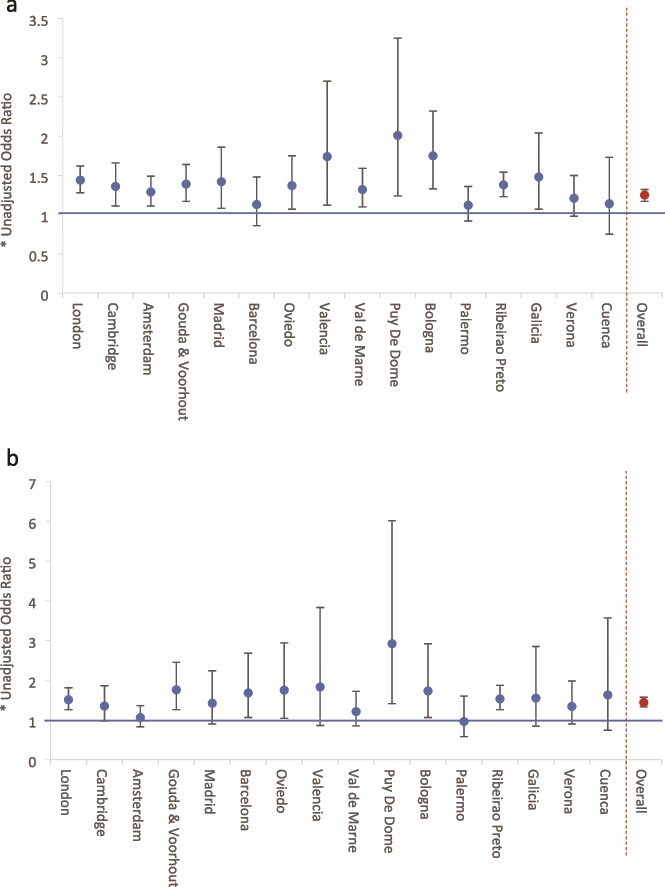
(a) Adversity Score by Site (See Supplementary Table S8a for Full Data). Note (1) odds ratios express the estimated increase in odds of psychosis for each additional adversity. Note (2) odds ratios range from 1.12 (Palermo) to 2.01 (Puy de Dôme), indicating an increase in odds of psychosis of around 10%-100% for each additional adversity. Note (3) interaction by site: *F* 1.07, *P* .380. (b) Severe adversity score by site (see Supplementary Table S8b for full data). Note (1) odds ratios express the estimated increase in odds of psychosis for each additional moderate or marked adversity. Note (2) odds ratios range from 0.96 (Palermo) to 2.92 (Puy de Dôme), indicating an increase in odds of psychosis of around 10%- 100% for each additional adversity. Note (3) interaction by site: *F* 1.17, *P* .287.

There were marked variations across the sites in the proportion of cases and controls reporting any adversity and modest variations in the estimated effect of any adversity, 3 or more adversities, and a count of adversities on odds of psychosis (Figure 3; Supplementary Table S8a and S8b). For controls, the proportion reporting any adversity ranged from 55% in Valencia, Spain, to 94% in London, UK. For cases, the proportion reporting any adversity ranged from 60% in Cuenca, Spain, to 97% in London, UK and 100% (*n*, 14) in Puy De Dome, France. Further, in unadjusted models, odds ratios varied widely from 0.4 in Cuenca to 12.1 in Madrid, with most falling between around 1.5 and 4.0. When we fit interaction terms for adversity by site to a logistic regression model, there was weak evidence that this variation was greater than expected by chance at *P* .05 (F 1.66, *P* .056). However, in some sites, samples were small and confidence intervals were wide, indicating a lack of precision in estimates. Similar patterns were evident for 3 or more adversities; however, when we modeled the number of adversities as a count variable, variation across sites was less marked (ie, unadj. ORs from 1.12 to 2.01; test for interaction: *F* 1.07, *P* .380). Further, these are unadjusted odds ratios. It was not possible, given the number of adversities, the number of sites, and the sample sizes in some sites, to provide meaningful and comparable fully adjusted odds ratios by site or estimates of the prevalence and effects of specific forms of adversity by site.

**Figure 4:**
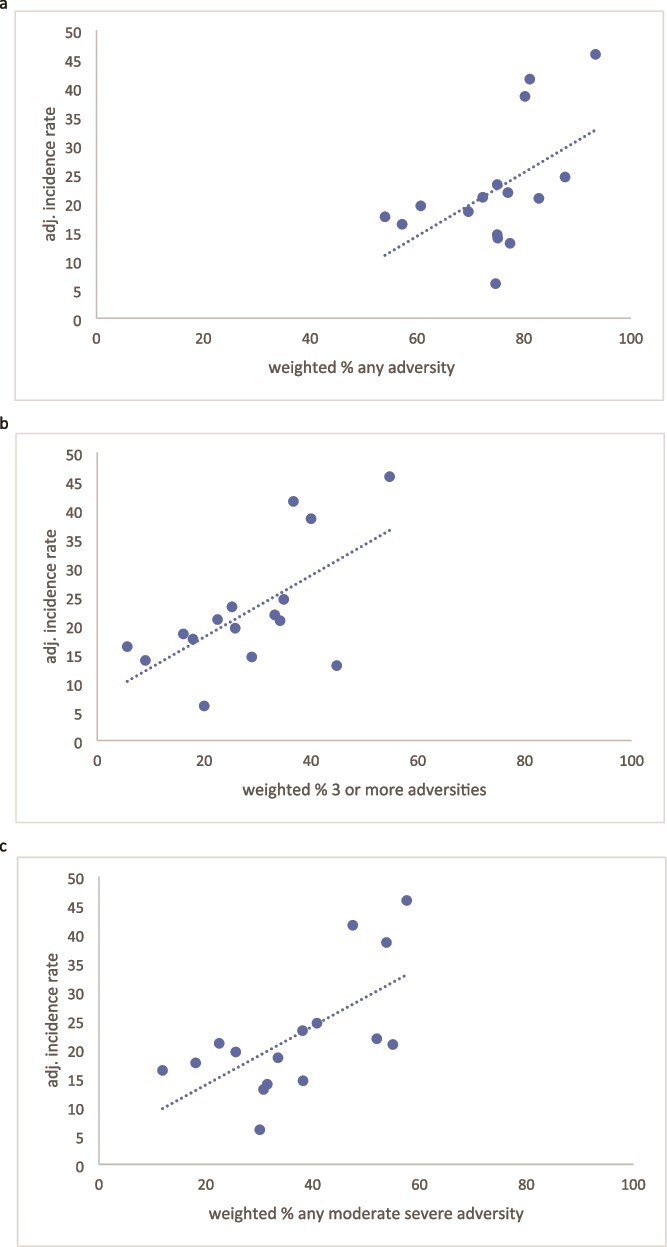
(a) Scatter plot: Incidence rates by percentage of controls reporting any adversity. (1) Spearman’s rank order correlation: rho 0.55 (*P* .0029). (2) Pearson’s correlation: *r* 0.53 (*P* .034). b. Scatter plot: Incidence rates by percentage of controls reporting 3 or more adversities. (1) Spearman’s rank order correlation: rho 0.56 (*P* .025) (2) Pearson’s correlation: *r* 0.64 (*P* .007). (c) Scatter plot: Incidence rates by percentage of controls reporting any moderate or marked adversity. (1) Spearman’s rank order correlation: rho 0.64 (*P* .009). (2) Pearson’s correlation: *r* 0.65 (*P* .007).

In EU-GEI, we observed an 8-fold difference in incidence rates across sites.^45^ Tentatively, we found some evidence that incidence rates tended to be higher in areas with a high proportion reporting any, multiple, and severe adversities (eg, 3 or more adversities: Spearman’s rank order correlation 0.56, *P* .025) (Figure 4; Supplementary Table S9). For example, the sites with the highest incidence (London, adj. IR 45.8 per 100 000; Val De Marne, adj. IR 41.5 per 100 000; Amsterdam, adj. IR 38.5 per 100 000) had the highest weighted proportions of controls reporting any, multiple, and severe adversities (eg, any moderate severe adversity: London, 58%; Val de Marne 48%; Amsterdam, 54%). This noted, there were also several sites with high weighted proportions reporting adversities and relatively low incidence rates (eg, Cambridge [UK] and Palermo [Italy]).

### By Severity, Frequency, and Age of Exposure (H5)

We next narrowed our focus to 5 forms of adversity on which we collected more detailed information concerning severity, frequency, and age at first exposure (household discord, psychological abuse, physical abuse, sexual abuse, and bullying).

In unadjusted analyses, we found that the odds of psychosis tended to be greatest for the most severe levels of exposure (Table 1). That is, compared with controls, cases were around 2-3 times more likely to report witnessing domestic violence and experiencing physical and sexual abuse and bullying of marked severity. By contrast, for other levels of severity, odds ratios were more modest (ie, Moderate, ORs < 2.0; Some, ORs < 1.4). For psychological abuse, odds ratios were around 2 for all levels of severity. In fully adjusted models, these patterns and effects remained, with minimal attenuation, for each form of adversity, with the exception of physical abuse. For physical abuse, odds ratios reduced to around 1.3. Considered together, there was a clear trend of increasing odds of psychosis with increasing severity, ie, adj. OR for exposure to any of the 5 adversities: Mild, 1.22 (95% CI 0.89-1.68); Moderate: 1.81 (95% CI 1.41-2.33); Marked: 2.59 (95% CI 1.92-3.49).

**Table 1:** Childhood Adversities and Psychotic Disorder, by Severity

**Adversity**		**Controls *n* = 1497 *n*^a^ (%)**		**Cases *n* = 1071 *n*^a^ (%)**		**OR (1)**	**95% CI**	***P***	**OR (2)**	**95% CI**	***P***	**OR (3)**	**95% CI**	***P***
Household discord														
	None	925	(63.1)	536	(52.0)	1.00	–	–	1.00	–	–	1.00	–	–
	Some	116	(7.9)	90	(8.7)	1.25	0.92-1.69	0.149	1.25	0.91-1.71	0.161	1.26	0.88-1.80	.212
	Moderate	180	(12.3)	128	(12.4)	1.19	0.92-1.54	0.179	1.21	0.93-1.59	0.158	1.37	1.01-1.86	.044
	Marked	141	(9.6)	126	(12.2)	1.57	1.20-2.04	0.001	1.58	1.20-2.09	0.001	1.65	1.95-2.27	.002
	Domestic violence	104	(7.1)	150	(14.6)	2.59	1.96-3.41	<0.001	2.61	1.95-3.50	<0.001	2.32	1.65-3.27	<.001
Psychological abuse														
	None	1360	(91.6)	858	(83.6)	1.00	–	–	1.00	–	–	1.00	–	
	Some	29	(2.0)	46	(4.5)	2.48	1.54-4.01	<0.001	2.51	1.53-4.13	<0.001	2.48	1.40-4.40	.002
	Moderate	64	(4.3)	83	(8.1)	2.09	1.49-2.93	<0.001	2.19	1.54-3.11	<0.001	1.73	1.14-2.63	.010
	Marked	31	(2.1)	39	(3.8)	2.15	1.33-3.45	0.002	2.20	1.32-3.66	0.002	1.89	1.04-3.46	.038
Physical abuse														
	None	1176	(79.3)	743	(72.1)	1.00	–	–	1.00	–	–	1.00	–	–
	Some	180	(12.1)	124	(12.0)	1.12	0.87-1.45	0.375	0.99	0.76-1.30	0.968	0.86	0.63-1.18	.350
	Moderate	108	(7.3)	132	(12.8)	1.96	1.49-2.59	<0.001	1.76	1.31-2.36	<0.001	1.37	0.96-1.94	.081
	Marked	20	(1.4)	31	(3.0)	2.42	1.35-4.32	0.003	1.87	1.02-3.44	0.042	1.32	0.65-2.69	.445
Sexual abuse														
	None	1376	(92.7)	912	(89.5)	1.00	–	–	1.00	–	–	1.00	–	–
	Some	50	(3.4)	43	(4.2)	1.29	0.85-1.96	0.230	1.56	1.00-2.41	0.048	1.50	0.90-2.50	.117
	Moderate	35	(2.4)	27	(2.7)	1.19	0.72-1.98	0.499	1.44	0.85-2.44	0.176	1.34	0.72-2.51	.355
	Marked	23	(1.6)	37	(3.6)	2.41	1.41-4.10	0.001	2.45	1.40-4.29	0.002	1.75	0.91-3.33	.091
Bullying														
	None	1045	(71.8)	594	(58.6)	1.00	–	–	1.00	–	–	1.00	–	–
	Some	174	(12.0)	129	(12.7)	1.34	1.04-1.73	0.024	1.41	1.08-1.84	0.012	1.55	1.14-2.12	.005
	Moderate	182	(12.5)	206	(20.3)	2.03	1.61-2.56	<0.001	1.91	1.50-2.43	<0.001	2.03	1.52-2.71	<.001
	Marked	55	(3.8)	85	(8.4)	2.78	1.93-4.02	<0.001	2.40	1.63-3.52	<0.001	2.29	1.47-3.57	<.001
Any^b^														
	None	572	(40.5)	265	(28.1)	1.00	–	–	1.00	–	–	1.00	–	–
	Some	228	(16.2)	128	(13.6)	1.24	0.95-1.61	0.114	1.26	0.96-1.66	0.102	1.22	0.89-1.68	.209
	Moderate	436	(30.9)	321	(34.1)	1.67	1.35-2.05	<0.001	1.68	1.35-2.09	<0.001	1.81	1.41-2.33	<.001
	Marked	175	(12.4)	228	(24.2)	2.99	2.34-3.82	<0.001	2.88	2.22-3.72	<0.001	2.59	1.92-3.49	<.001

OR (1) Unadjusted

OR (2) Adjusted for age, sex, and ethnicity

OR (3) Adjusted for age, sex, ethnicity, IQ, parental history of psychosis, and parental social class at birth.

a *n* is the number with complete data.

b Any adversity: one or more adversity at the specific level of severity but not greater; for household discord, marked and moderate included in moderate category for any adversity and domestic violence including in marked category for any adversity.

Similar, but slightly weaker, patterns were evident for frequency of exposure, ie, a general tendency for higher odds of psychosis for frequent (monthly) exposure relative to infrequent (less than monthly) exposure (Supplementary Table S10), with the possible exception of bullying. The fully adjusted odds ratio was 2.10 (95% CI 1.67-2.66) for frequent exposure to any of the 5 adversities compared with 1.39 (95% CI 1.01-1.93) for infrequent exposure.

Finally, there was some evidence that the effects of each adversity varied by age of first exposure (Supplementary Table S11). For household discord, psychological abuse, and sexual abuse, earlier age of first exposure (0-11 years) was associated with modestly greater increased odds of psychosis than later exposure (12-16 years). For example, early first exposure to sexual abuse (age of 11 and younger) was associated with a near 2-fold increased odds of psychosis (adj. OR 1.88, 95% CI 1.22-2.89) and later first exposure (age 12 and older) was associated with, at most, a modest ~50% increased odds (adj. OR 1.45, 95% CI 0.82-2.57). For bullying—and to a lesser extent for physical abuse—the opposite was observed, that is, a greater effect for first exposure during adolescence (ie, 11 or younger: adj. OR 1.81, 95% CI 1.40-2.34; 12 or older: adj. OR 2.25, 95% CI 1.66-3.04).

## Discussion

Our findings deepen our understanding of the relationship between childhood adversities and psychosis in several novel and important ways. In addition to replicating previous studies (H1, H2), we found: the frequency and magnitude of effects of each adversity varied (H1, H2); effects of some adversities varied by sex (H3); the magnitude of overall effects and the prevalence of adversities in source populations varied by place, with tentative but novel evidence that these variations were positively correlated with the incidence of psychosis (H4); and effects varied by severity, frequency, and, to a lesser extent, age of exposure for some of the 5 sub-types of adversity examined in more detail (H5).

### Methodological Considerations

There are several methodological issues to consider. Recent research has found that prospective and retrospective assessments of childhood adversity identify different groups of people.^48^ This illustrates the challenge of accurately capturing exposure to childhood adversities and the potential for current mental state to influence retrospective recall of past experiences. This is especially problematic for case–control studies as cases may be more likely to recall past adversities than controls. In addressing this, others have pointed to the consistency in reported associations between adversity and psychosis, irrespective of study design.^6^ In this study, we collected information on adversities using a well-validated semi-structured interview, using life interview techniques, to elicit concrete descriptions of experiences that were rated, conservatively, by trained researchers using standard criteria. Further, we found that the odds ratios were highest for the most severe experiences. These are profound, deeply traumatic, and unambiguous experiences that are unlikely to be forgotten or only recalled in light of subsequent outcomes.

A similar issue concerns control recruitment. In each setting, we sought to recruit control samples that were representative of the source population from which cases were identified. To achieve this, a mixture of random and quota sampling was used, with the specific approach tailored to what was feasible in each setting. However, it is possible that individuals who had experienced past adversities were less likely to take part, consequently underestimating the prevalence of adversities in source populations. We used post-stratification weights to account for selection bias related to age, sex, and ethnic group, but this only partially addresses this issue. Further, in settings where the number of controls was small, estimates of the prevalence of adversities in source populations are less precise. Caution is therefore needed in interpreting setting-specific findings and in drawing inferences about connections between the prevalence of adversities in a population and rates of psychosis.

In final models, we adjusted for several potential confounders, including household socio-economic position at birth (parental social class), cognition (IQ), and genetic risk (history of parental psychosis). These are imperfect measures that may not fully account for confounding by these variables. This noted, previous studies that have used more comprehensive measures of these constructs have not found evidence of substantial confounding, for example, gene–adversity correlation.^9^ We did not adjust for cannabis use and adult adversity and socio-economic position as these may be on causal pathways between childhood adversity and psychosis. However, it is also possible that use of cannabis early in adolescence may be associated with subsequent experiences of adversity within our period of observation (ie, before age 17) and, as such, partly confound the observed associations between adversities and psychosis. Our choice may therefore underadjust estimates, adding an important consideration in the interpretation of our findings, especially where odds ratios are modest (ie, <1.5; eg, physical abuse).

### Childhood Adversities and Psychosis (1)

These considerations noted, the weight of evidence is that childhood adversities are associated, to varying extents, with an increased risk of psychosis, particularly when these cluster.^2^^,^^4^^,^^6^^,^^23^ This constitutes an important insight into the developmental backgrounds against which psychosis emerges for many, that is, backgrounds characterized by exposure to several interconnected adversities spanning multiple levels and domains: neighbourhood, household, school, and peer relationships.

We further found some variations by sex. In line with some previous reports (eg,^26^^,^^27^^,^^49^), we found that women were more likely to report physical and sexual abuse and that odds ratios were greater for women than for men. A recent meta-analysis of 183 studies reported an overall odds ratio for sexual abuse of 2.7 for women and 1.8 for men,^6^ strikingly similar to our findings (ie, 2.6 vs. 1.9). There are several plausible mechanisms that may account for these differences (eg, experiences are more threatening for girls/women), and these may account for some of the differences by sex in presentation (eg, more affective and positive symptoms).^20^^,^^25^ Intriguingly, we also found that neglect was more strongly associated with odds of psychosis in men. Prachason et al,^49^ in their study of childhood adversities and psychopathology in a general population sample, found that neglect was associated with a range of symptoms, including paranoia, specifically in men. Further, in their recent review, Comacchio et al^25^ found that childhood abuse was more often associated with affective and positive symptoms among women and more often associated with negative symptoms and poorer cognitive performance among men. These findings suggest variations by sex in the prevalence and effects of different adversities may contribute to some of the differences by sex in the manifestations of psychosis. This noted, previous findings on differences by sex are mixed^6^ and there is consequently a need for some caution. Further, we did not find any variations by diagnosis; however, this may reflect our use of broad diagnostic categories and more refined analyses by symptom dimensions may be more informative.^18^

### Childhood Adversities and Psychosis (2)

Well-established variations in rates of psychosis by place, social and ethnic group, and over time^50^ may reflect variations in the distribution and effects of causal factors in specific populations at particular times. In this study, we found that the distribution and effects of adversities varied by setting and that there was a modest positive correlation between the prevalence of adversities and the incidence of psychosis in each setting. We found similar patterns and associations in our analyses of EU-GEI socioeconomic deprivation and cannabis data^33^^,^^45^^,^^51^; together, these findings suggest that environments that act as reservoirs of greater social adversity shape population-level rates of psychosis.

There was variation in the magnitude of odds ratios by type of adversity. Crudely, odds ratios were small to modest (ie, <1.5) for adversities related to household deprivation and upheaval (eg, money problems, parental separation, change of school) and modest to strong (ie, >1.5) for adversities related to maltreatment and threat (eg, psychological and sexual abuse, bullying), with the exception of physical abuse. Further reinforcing this, in more fine-grained analyses, we found that severe and frequent adversities were most strongly associated with psychosis, with some indications that effects for some adversities depended on age of first exposure. To make this concrete, on the CECA severity scale, the highest rating captures experiences that involve extreme levels of threat, hostility, and violence, including assault and injury; coercion, humiliation, and cruelty; and threat to life. One in 4 of those with a psychotic disorder in our samples reported at least 1 adversity at this level of severity compared with just over 1 in 10 in our control samples. These findings broadly replicate what we found in a smaller sample in London (UK)^28^ and align with findings from other studies that suggest risk of psychosis is especially high among those who experience the most extreme forms of sexual abuse^52^ and among those who, in adulthood, experience discrimination,^53^ threatening life events,^54^ and hostile family environments,^53^^,^^55^ and who live in areas with high levels of crime, social fragmentation, and poverty.^56^ There are plausible mechanisms via which these adversities may increase risk. Persistent exposure to threat and uncertainty may impact on physiological,^57^ immune,^58^ and emotional^59^ responses and on cognitive processes and schema^15^ in ways that establish vulnerabilities to psychosis. For example, at a psychological level, intense and chronic experiences of threat may lead to anxiety, helplessness, dissociative experiences, and the development of tendencies to see or anticipate threat in neutral situations (ie, paranoia)—and, ultimately, to the development of affective symptoms, hallucinations, and persecutory delusions.^11^^,^^13^^,^^21^

Others have adopted a different approach and sought to aggregate adversities into a single construct (eg, part of the “exposome”).^60^ This approach is valuable in summarizing the overall effect of adversities on risk of psychosis and can provide a simple metric capturing environmental exposures for use in studies of mechanisms, interactions, and prediction. We did this to an extent in creating an index of adversities and examining cumulative effects. However, our more detailed analyses suggest that critical nuances may be lost in this approach, with the consequence that findings may be misleading and of limited value in informing targeted prevention strategies and interventions. Another alternative approach that may further clarify how adversities cluster to increase risk is latent class analyses, which can be used to identify sub-populations characterized by distinct probabilities of exposure to a range of risks. However, as far as we are aware, no such studies of adversities and psychotic disorders have been reported.

### Implications

There may be multiple causal pathways to the development of psychosis, and these may include a sociodevelopmental pathway in which exposures to social adversities across the life course are the salient causes.^2^^,^^61^ Our findings elaborate on this, suggesting some specificity for adversities involving threat, hostility, and violence, especially when occurring at developmentally critical stages, and tentatively indicate that eco-social contexts or niches characterised by high levels of threat, social fragmentation, isolation, and substance use may underlie high rates of psychosis in some populations. This is speculative. The association we observed between the prevalence of adversities and rates of psychosis was modest, at most, with considerable uncertainty in estimates in some settings because of small sample sizes. This said, given that any pathway to psychosis likely involves complex interactions between an array of causal factors, associations between the prevalence of any given risk factor and population rates of psychosis are likely to be modest. Further research is needed to test, refine, and challenge the tentative implications proposed here.

These caveats noted, our findings illustrate the importance of context-specific data on the prevalence and impacts of risks. Focusing on how particular risk and protective factors combine and interact in specific historical and social contexts is of considerable value for better understanding the nature and determinants of psychoses and for developing effective strategies for prevention, service delivery, and intervention tailored to local settings. For example, in ecological contexts where there are high levels of disadvantage and adversity, irrespective of causal connections, many more who present to services with a psychotic disorder will do so against a background of poverty and developmental trauma. This points to the need, in such contexts, for more services and interventions tailored to these challenges, to the lived experiences of trauma that many with psychosis have endured.

## Supplementary Material

Supplementary_materials_sbag030

## References

[1] Varese F, Smeets F, Drukker M, et al. Childhood adversities increase the risk of psychosis: a meta-analysis of patient-control, prospective- and cross-sectional cohort studies. *Schizophr Bull*. 2012;38:661-671. 10.1093/schbul/sbs050 22461484 PMC3406538

[2] Morgan C, Gayer-Anderson C. Childhood adversities and psychosis: evidence, challenges, implications. *World Psychiatry*. 2016;15:93-102. 10.1002/wps.20330 27265690 PMC4911761

[3] Pastore A, De Girolamo G, Tafuri S, Tomasicchio A, Margari F. Traumatic experiences in childhood and adolescence: a meta-analysis of prospective studies assessing risk for psychosis. *Eur Child Adolesc Psychiatry*. 2022;31:215-228. 10.1007/s00787-020-01574-9 32577908

[4] McKay MT, Cannon M, Chambers D, et al. Childhood trauma and adult mental disorder: a systematic review and meta-analysis of longitudinal cohort studies. *Acta Psychiatr Scand*. 2021;143:189-205. 10.1111/acps.13268 33315268

[5] Arango C, Dragioti E, Solmi M, et al. Risk and protective factors for mental disorders beyond genetics: an evidence-based atlas. *World Psychiatry*. 2021;20:417-436. 10.1002/wps.20894 34505386 PMC8429329

[6] Zhou L, Sommer IEC, Yang P, et al. What do four decades of research tell us about the association between childhood adversity and psychosis: an updated and extended multi-level meta-analysis. *Am J Psychiatry*. 2025;182:360-372. 10.1176/appi.ajp.20240456 40165558

[7] Morgan C, Reininghaus U, Fearon P, et al. Modelling the interplay between childhood and adult adversity in pathways to psychosis: initial evidence from the AESOP study. *Psychol Med*. 2014;44:407-419. 10.1017/S0033291713000767 23590972 PMC4081841

[8] Morgan C, Reininghaus U, Reichenberg A, Frissa S, Hotopf M, Hatch SL. Adversity, cannabis use and psychotic experiences: evidence of cumulative and synergistic effects. *Br J Psychiatry*. 2014;204:346-353. 10.1192/bjp.bp.113.134452 24627297 PMC4006086

[9] Woolway GE, Smart SE, Lynham AJ, et al. Schizophrenia polygenic risk and experiences of childhood adversity: a systematic review and meta-analysis. *Schizophr Bull*. 2022;48:967-980. 10.1093/schbul/sbac049 35674151 PMC9434424

[10] Aas M, Alameda L, Di Forti M, et al. Synergistic effects of childhood adversity and polygenic risk in first-episode psychosis: the EU-GEI study. *Psychol Med*. 2023;53:1970-1978. 10.1017/S0033291721003664 37310339 PMC10106300

[11] Williams J, Bucci S, Berry K, Varese F. Psychological mediators of the association between childhood adversities and psychosis: a systematic review. *Clin Psychol Rev*. 2018;65:175-196. 10.1016/j.cpr.2018.05.009 30243100

[12] Sideli L, Murray RM, Schimmenti A, et al. Childhood adversity and psychosis: a systematic review of bio-psycho-social mediators and moderators. *Psychol Med*. 2020;50:1761-1782. 10.1017/S0033291720002172 32624020

[13] Alameda L, Rodriguez V, Carr E, et al. A systematic review on mediators between adversity and psychosis: potential targets for treatment. *Psychol Med*. 2020;50:1966-1976. 10.1017/S0033291720002421 32744193

[14] Grady S, Twomey C, Cullen C, Gaynor K. Does affect mediate the relationship between interpersonal trauma and psychosis? A systematic review and meta-analysis. *Schizophr Res*. 2024;264:435-447. 10.1016/j.schres.2024.01.008 38245930

[15] Croft J, Martin D, Madley-Dowd P, et al. Childhood trauma and cognitive biases associated with psychosis: a systematic review and meta-analysis. *PLoS One*. 2021;16:e0246948. 10.1371/journal.pone.0246948 33630859 PMC7906349

[16] Cancel A, Dallel S, Zine A, El-Hage W, Fakra E. Understanding the link between childhood trauma and schizophrenia: a systematic review of neuroimaging studies. *Neurosci Biobehav Rev*. 2019;107:492-504. 10.1016/j.neubiorev.2019.05.024 31163206

[17] Alameda L, Liu Z, Sham PC, et al. Exploring the mediation of DNA methylation across the epigenome between childhood adversity and first episode of psychosis-findings from the EU-GEI study. *Mol Psychiatry*. 2023;28:2095-2106. 10.1038/s41380-023-02044-9 37062770

[18] Alameda L, Christy A, Rodriguez V, et al. Association between specific childhood adversities and symptom dimensions in people with psychosis: systematic review and meta-analysis. *Schizophr Bull*. 2021;47:975-985. 10.1093/schbul/sbaa199 33836526 PMC8266673

[19] Grindey A, Bradshaw T. Do different adverse childhood experiences lead to specific symptoms of psychosis in adulthood? A systematic review of the current literature. *Int J Ment Health Nurs*. 2022;31:868-887. 10.1111/inm.12992 35306711

[20] Bailey T, Alvarez-Jimenez M, Garcia-Sanchez AM, Hulbert C, Barlow E, Bendall S. Childhood trauma is associated with severity of hallucinations and delusions in psychotic disorders: a systematic review and meta-analysis. *Schizophr Bull*. 2018;44:1111-1122. 10.1093/schbul/sbx161 29301025 PMC6101549

[21] Bloomfield MAP, Chang T, Woodl MJ, et al. Psychological processes mediating the association between developmental trauma and specific psychotic symptoms in adults: a systematic review and meta-analysis. *World Psychiatry*. 2021;20:107-123. 10.1002/wps.20841 33432756 PMC7801841

[22] Ajnakina O, Trotta A, Oakley-Hannibal E, et al. Impact of childhood adversities on specific symptom dimensions in first-episode psychosis. *Psychol Med*. 2016;46:317-326. 10.1017/S0033291715001816 26383785

[23] Saetren SS, Bjornestad JR, Ottesen AA, et al. Unraveling the concept of childhood adversity in psychosis research: a systematic review. *Schizophr Bull*. 2024;50:1055-1066. 10.1093/schbul/sbae085 38811352 PMC11349006

[24] Kim B, Royle M. Annual research review: mapping the multifaceted approaches and impacts of adverse childhood experiences - an umbrella review of meta-analyses. *J Child Psychol Psychiatry*. 2025;66:399-416. 10.1111/jcpp.14022 38772385

[25] Comacchio C, Lasalvia A, Ruggeri M. Current evidence of childhood traumatic experiences in psychosis - focus on gender differences. *Psychiatry Res*. 2019;281:112507. 31465988 10.1016/j.psychres.2019.112507

[26] Fisher H, Morgan C, Dazzan P, et al. Gender differences in the association between childhood abuse and psychosis. *Br J Psychiatry*. 2009;194:319-325. 10.1192/bjp.bp.107.047985 19336782

[27] Gayer-Anderson C, Fisher HL, Fearon P, et al. Gender differences in the association between childhood physical and sexual abuse, social support and psychosis. *Soc Psychiatry Psychiatr Epidemiol*. 2015;50:1489-1500. 10.1007/s00127-015-1058-6 PMC4589555 25893995

[28] Morgan C, Gayer-Anderson C, Beards S, et al. Threat, hostility and violence in childhood and later psychotic disorder: population-based case–control study. *Br J Psychiatry*. 2020;217:575-582. 10.1192/bjp.2020.133 32778182 PMC7525109

[29] Gayer-Anderson C, Morgan C. Social networks, support and early psychosis: a systematic review. *Epidemiol Psychiatr Sci*. 2012;22:131-146. 10.1017/S2045796012000406 22831843 PMC6998121

[30] McGrath JJ, McLaughlin KA, Saha S, et al. The association between childhood adversities and subsequent first onset of psychotic experiences: a cross-national analysis of 23 998 respondents from 17 countries. *Psychol Med*. 2017;47:1230-1245. 10.1017/S0033291716003263 28065209 PMC5590103

[31] Sidebotham P, Heron J, Golding J, team As. Child maltreatment in the "children of the nineties:" deprivation, class, and social networks in a UK sample. *Child Abuse Negl*. 2002;26:1243-1259. 12464299 10.1016/s0145-2134(02)00415-5

[32] Krieger N . Ecosocial Theory, Embodied Truths, and the people's Health. Small Books, Big Ideas in Population Health 4. New York, NY: Oxford University Press, 2021. 1.

[33] Di Forti M, Quattrone D, Freeman TP, et al. The contribution of cannabis use to variation in the incidence of psychotic disorder across Europe (EU-GEI): a multicentre case-control study. *Lancet Psychiatry*. 2019;6:427-436. 10.1016/S2215-0366(19)30048-3 PMC7646282 30902669

[34] Gayer-Anderson C, Jongsma HE, Di Forti M, et al. The EUropean network of National Schizophrenia Networks Studying Gene-Environment Interactions (EU-GEI): incidence and first-episode case-control Programme. *Soc Psychiatry Psychiatr Epidemiol*. 2020;55:645-657. 10.1007/s00127-020-01831-x 31974809

[35] Bifulco A, Brown GW, Harris TO. Childhood experience of care and abuse (CECA): a retrospective interview measure. *J Child Psychol Psychiatry*. 1994;35:1419-1435. 7868637 10.1111/j.1469-7610.1994.tb01284.x

[36] Bifulco A, Bernazzani O, Moran PM, Jacobs C. The childhood experience of care and abuse questionnaire (CECA.Q): validation in a community series. *Br J Clin Psychol*. 2005;44:563-581. 10.1348/014466505X35344 16368034

[37] Mallett R . MRC Sociodemographic Schedule. Institute of Psychiatry, 1997.

[38] Harrison E, Rose D. The European Socio-Economic Classification (ESeC) User Guide. Colchester, UK: Institute for Social and Economic Research, 2006.

[39] McGuffin P, Farmer A, Harvey I. A polydiagnostic application of operational criteria in studies of psychotic illness. Development and reliability of the OPCRIT system. *Arch Gen Psychiatry*. 1991;48:764-770. 1883262 10.1001/archpsyc.1991.01810320088015

[40] Initiative NG . Family Interview for Genetic Studies (FIGS). Rockville, MD: National Institute of Mental Health, 1991.

[41] Wechsler D . WAIS-III, Wechsler adult intelligence scale: administration and scoring manual. *Psychological Corporation*. 1997.

[42] Sterne JA, White IR, Carlin JB, et al. Multiple imputation for missing data in epidemiological and clinical research: potential and pitfalls. *BMJ*. 2009;338:b2393. 10.1136/bmj.b2393 19564179 PMC2714692

[43] Little R, Riubin D. Statistical Analyses with Missing Data2nd edn. New York: Wiley, 2002.

[44] White IR, Royston P, Wood AM. Multiple imputation using chained equations: issues and guidance for practice. *Stat Med*. 2011;30:377-399. 10.1002/sim.4067 21225900

[45] Jongsma HE, Gayer-Anderson C, Lasalvia A, et al. Treated incidence of psychotic disorders in the multinational EU-GEI study. *JAMA Psychiatry*. 2018;75:36-46. 10.1001/jamapsychiatry.2017.3554 29214289 PMC5833538

[46] Valliant R, Dever JA. Survey Weights: A Step-by-Step Guide to Calculation. College Station, TX: Stata Press, 2018.

[47] Stata. STATA Statistical Software, Release 8. College Station, TX: Stata Corporation; 2003.

[48] Baldwin JR, Coleman O, Francis ER, Danese A. Prospective and retrospective measures of child maltreatment and their association with psychopathology: a systematic review and meta-analysis. *JAMA Psychiatry*. 2024;81:769-781. 10.1001/jamapsychiatry.2024.0818 38691376 PMC11063927

[49] Prachason T, Mutlu I, Fusar-Poli L, et al. Gender differences in the associations between childhood adversity and psychopathology in the general population. *Soc Psychiatry Psychiatr Epidemiol*. 2024;59:847-858. 10.1007/s00127-023-02546-5 37624463 PMC11087312

[50] Jongsma HE, Turner C, Kirkbride JB, Jones PB. International incidence of psychotic disorders, 2002-17: a systematic review and meta-analysis. *Lancet Public Health*. 2019;4:e229-e244. 10.1016/S2468-2667(19)30056-8 31054641 PMC6693560

[51] Brink V, Andleeb H, Gayer-Anderson C, et al. The role of social deprivation and cannabis use in explaining variation in the incidence of psychotic disorders: findings from the EU-GEI study. *Schizophr Bull*. 2024;50:1039-1049. 10.1093/schbul/sbae072 38788048 PMC11349009

[52] Bebbington PE, Bhugra D, Brugha T, et al. Psychosis, victimisation and childhood disadvantage: evidence from the second British National Survey of psychiatric morbidity. *Br J Psychiatry*. 2004;185:220-226. 15339826 10.1192/bjp.185.3.220

[53] Misra S, Gelaye B, Williams DR, et al. Perceived major experiences of discrimination, ethnic group, and risk of psychosis in a six-country case-control study. *Psychol Med*. 2021;52:3668-3676. 10.1017/S0033291721000453 33648622

[54] Beards S, Fisher HL, Gayer-Anderson C, et al. Threatening life events and difficulties and psychotic disorder. *Schizophr Bull*. 2020;46:814-822. 10.1093/schbul/sbaa005 32047940 PMC7342097

[55] Varchmin L, Montag C, Treusch Y, Kaminski J, Heinz A. Traumatic events, social adversity and discrimination as risk factors for psychosis - an umbrella review. *Front Psych*. 2021;12:665957. 10.3389/fpsyt.2021.665957 PMC8569921 34744806

[56] Bhavsar V, Boydell J, Murray R, Power P. Identifying aspects of neighbourhood deprivation associated with increased incidence of schizophrenia. *Schizophr Res*. 2014;156:115-121. 10.1016/j.schres.2014.03.014 24731617

[57] Tosato S, Tomassi S. The biological correlates of childhood trauma in first episode psychosis. *J Psychopathol*. 2020;20:70-76.

[58] Radhakrishnan R, Kaser M, Guloksuz S. The link between the immune system, environment, and psychosis. *Schizophr Bull*. 2017;43:693-697. 10.1093/schbul/sbx057 28969353 PMC5472105

[59] Muddle S, Jones B, Taylor G, Jacobsen P. A systematic review and meta-analysis of the association between emotional stress reactivity and psychosis. *Early Interv Psychiatry*. 2022;16:958-978. 10.1111/eip.13247 34904353

[60] Guloksuz S, van Os J, Rutten BPF. The exposome paradigm and the complexities of environmental research in psychiatry. *JAMA Psychiatry*. 2018;75:985-986. 10.1001/jamapsychiatry.2018.1211 29874362

[61] Morgan C, Charalambides M, Hutchinson G, Murray RM. Migration, ethnicity, and psychosis: toward a sociodevelopmental model. *Schizophr Bull*. 2010;36:655-664. 10.1093/schbul/sbq051 20513653 PMC2894585

