# Supplementary material for: Childhood Adversities and Psychosis Across Populations: Insights From the 6-Country EU-GEI Study
Source: Schizophr Bull. 2026 May 4;52(3):sbag030. doi: 10.1093/schbul/sbag030 (PMC13137326; doi:10.1093/schbul/sbag030)
Supplement: Supplementary_materials_sbag030 [file supplementary_materials_sbag030.docx]

**Supplementary Materials**

**Appendix 1:** Ethics committees

**Appendix 2:** List of childhood adversities

**Appendix 3:** Severity rating scales

**Supplementary Table 1.** Control sample compared with the population-at-risk by catchment area.

**Supplementary Table 2a.** Sociodemographic and clinical characteristics by case-control status.

**Supplementary Table 2b.** Adversities (preset vs. absent) by case-control status.

**Supplementary Table 3.** Main effects, each adversity (present vs. absent).

**Supplementary Table 4.** Associations between each childhood adversity.

**Supplementary Table 5.** Number of adversities, index.

**Supplementary Table 6.** Main effects, each adversity (present vs. absent) by sex.

**Supplementary Table 7.** Main effects for each type of childhood adversity, by diagnosis.

**Supplementary Table 8a.** Adversity (any present vs. none; 3 plus; score) by site.

**Supplementary Table 8b.** Moderate or Marked Adversity (any present vs. none; 3 plus; score) by site.

**Supplementary Table 9.** Incidence rates and prevalence of adversities in controls, by site.

**Supplementary Table 10.** Childhood adversities and psychotic disorder, by frequency.

**Supplementary Table 11.** Childhood adversities and psychotic disorder, by age of first exposure.

**Appendix 1:** Ethics committees

South London and Maudsley and Institute of Psychiatry Research Ethics Committee; National Research Ethics Service Committee East of England–East Cambridge; Medisch-Ethische Toetsingscommissie van het Academisch Centrum te Amsterdam; Comité Ético de Investigación Clínica Hospital Gregorio Marañón; Comité Ético de Investigación Clínica del Hospital Clinic de Barcelona; Comité Ético de Investigación Clínica del Hospital Clinic Universitari de Valencia; Comité Ética de la Investigación Clínica del Principado de Asturias; Comité Ético de Investigación Clínica de Galicia; Comité Ético de Investigación Clínica del Hospital Virgen de la Luz de Cuenca; Comité de Protéction des Personnes–CPP Île de France IX; Comitato Etico Policlinico S Orsola Malpighi; Comitato Etico Azienda Ospedaleria Universitaria di Verona; Comitato Etico Palermo 1, Azienda Ospedaliera Policlinico ‘Paolo Giaccone’; and Research Ethics Committee of the clinical Hospital of Ribeirão Preto Medical School, University of São Paulo, Brazil.

**Appendix 2:** List of childhood adversities

| Parent died | - Before the age of 17 did one or both of your parents die? |
| --- | --- |
| Separated, mother | - Before the age of 17 were you separated from your mother (longer than six months)? |
| Separated, father | - Before the age of 17 were you separated from your father (longer than six months)? |
| Change school | - Before the age of 17 years did you ever change schools (other than change from primary to secondary)? |
| Excluded | - Before the age of 17 years were you ever expelled from school? |
| In care | - Before the age of 17 years did you ever get taken into care (i.e., children’s home, fostered)? |
| Money problems | - Before the age of 17 years did you ever have any times when your family was significantly short of money? |
| Neglect | - Before the age of 17 years did were your basic needs (for food, clean clothing, etc.) ever neglected ? |
| Household Discord | - Before the age of 17 years were you ever aware of frequent arguments or extreme tensions between your parents? |
| Psychological Abuse | - Before the age of 17 years were you ever tormented or treated cruelly by a parent or a member of household? |
| Physical Abuse | - Before the age of 17 years were you ever hit or slapped on a number of occasions, sufficient to cause harm? |
| Sexual Abuse | - Before the age of 17 years were you ever have any unwanted sexual experiences? |
| Bullied | By bullying we mean when people of a similar age to you said mean and hurtful things or made fun of you or called you mean and hurtful names; completely ignore or excluded you from their grop of friends or left you out of things on purpose; hit, kicked, or shoved you, or locked you in a room; told lies or spread rumours about you; other hurtful things.   - Before the age of 17 years did you have any of these experiences? |
| Lonely | - Before the age of 17 years were you ever feel lonely for a significant period (i.e., 6 months or more)? |
| Peer confidant | - Before the age of 17 years were there others your own age you could go to with problems or to discuss feelings? |
| Adult confidant | - Before the age of 17 years were there any adults you could go to with problems or to discuss feelings? |

**Appendix 3.** Severity rating scales

(1) Household Discord

| 0 None | Absence of discord and tension or in very rare instances |
| --- | --- |
| 1 Some | Any rows, arguments or tension with low frequency, e.g. less than monthly |
| 2 Moderate | Frequent and/or intense arguments and tense atmosphere |
| 3 Marked | Rating of 2, plus people stop speaking to each other for days or weeks |
| 4 Violence | Occurrence of violence between parents on more than one occasion |

(2) Psychological Abuse

| 0 None | No abuse, or minimal |
| --- | --- |
| 1 Some | Single abusive incident or very low frequency and intensity |
| 2 Moderate | High frequency or pervasiveness of one type of abuse (e.g., humiliation) |
| 3 Marked | High frequency or pervasiveness of multiple types of abuse (e.g., humiliation, terrorising) |

(3) Physical Abuse

| 0 None | No abuse; Object used without possibility of causing injury; Pushed, grabbed or shoved |
| --- | --- |
| 1 Some | Single instances being slapped around head or face; Hit hard or repeatedly around body |
| 2 Moderate | Punched, kicked, bitten, burnt; Implement or object used that could have caused injury |
| 3 Marked | Life threatened; Severe/multiple injuries likely; Abuse intense and frequent |

(4) Sexual Abuse

| 0 None | No abuse; Flashed by stranger; Willing sexual contact with someone the same age |
| --- | --- |
| 1 Some | Single instances non-intercourse abuse; Forced to watch sex acts; Verbal solicitations |
| 2 Moderate | Repeated non-intercourse abuse (touching of own or others’ genitals) |
| 3 Marked | Repeated sexual abuse that involved intercourse |

(5) Bullying

| 0 None | No teasing or bullying, or minimal |
| --- | --- |
| 1 Some | Sporadic teasing; Some upset |
| 2 Moderate | Persistent teasing, but not 3; Physically hit, but not 3 (i.e., no bruising or injury) |
| 3 Marked | Physically hurt (e.g., bruised, cut, burnt); Intense verbal bullying |

**Supplementary Table 1.** Control sample compared with the population-at-risk by catchment area.

|  | Age | | Sex | | Ethnicity | |
| --- | --- | --- | --- | --- | --- | --- |
|  | 18-34 year olds | | Men | | Majority status | |
|  | Population | Controls | Population | Controls | Population | Controls |
|  | n (%) | n (%) | n (%) | n (%) | n (%) | n (%) |
| Southeast London | **214,713 (50.4)** | **155 (67.4)** | 212,918 (49.9) | 113 (49.1) | 175,706 (41.2) | 105 (45.7) |
| Cambridgeshire | 582,569 (37.5) | 37 (34.9) | 782,607 (50.4) | 50 (47.2) | 1,238,172 (79.7) | 85 (80.2) |
| Amsterdam | **256,659 (41.3)** | **53 (52.5)** | 313,287 (50.4) | 47 (46.5) | 293,709 (47.3) | 57 (56.4) |
| Gouda & Voorhout | 242,922 (31.2) | 38 (34.9) | 384,975 (50.2) | 52 (47.7) | 651,786 (85.0) | 103 (94.5) |
| Madrid | 149.670 (36.1) | 17 (44.7) | 205,364 (49.5) | 18 (47.4) | 329,424 (79.4) | 30 (79.0) |
| Barcelona | 311,664 (35.3) | 14 (37.8) | 426,259 (48.2) | 16 (43.2) | 299,983 (82.4) | 28 (75.7) |
| Valencia | 125,239 (34.4) | 11 (34.4) | 180,696 (49.6) | 15 (46.9) | 428,482 (92.6) | 26 (81.3) |
| Oviedo | **253,358 (30.8)** | **23 (59.0)** | 226,980 (49.1) | 19 (48.7) | 556,193 (96.7) | 33 (84.6) |
| Santiago | 189,135 (32.9) | 11 (29.7) | 286,767 (49.9) | 18 (47.4) | 160,723 (82.4) | 38 (100.0) |
| Cuenca | 68,672 (35.2) | 15 (39.5) | 102,695 (52.6) | 20 (52.6) | 179,220 (66.8) | 31 (81.6) |
| Val-de-Marne | **198,767 (38.9)** | **50 (50.0)** | 242,333 (47.5) | 47 (47.0) | **342,090 (67.0)** | **51 (51.0)** |
| Puy-de-Dome | **61,684 (27.2)** | **22 (46.8)** | 113,579 (50.1) | 21 (44.7) | 213,780 (94.4) | 47 (100.0) |
| Bologna | **272,987 (29.3)** | **43 (66.2)** | 455,829 (48.9) | 24 (36.9) | **727,700 (78.1)** | **60 (92.3)** |
| Veneto | **202,672 (40.1)** | **65 (56.5)** | 259,282 (51.3) | 53 (46.1) | 446,523 (88.3) | 103 (89.6) |
| Palermo | **541,472 (34.0)** | **64 (64.0)** | 781,004 (49.0) | 49 (49.0) | 1,493,856 (93.7) | 92 (92.0) |
| Ribeirão Preto | **1,219,115 (46.3)** | **216 (71.8)** | 1,299,112 (49.4) | 144 (47.7) | 1,745,638 (66.3) | 206 (68.2) |

**Note:** Bold indicates p < 0.05. However, this should be intereprted with caution, given p is so heavily influenced by sample size.

**Supplementary Table 2a.** Sociodemographic and clinical characteristics by case-control status.

|  | | Controls  n = 1,497 | | Cases  n = 1,071 | | t | df | p |
| --- | --- | --- | --- | --- | --- | --- | --- | --- |
| Age | |  |  |  |  |  |  |  |
|  | Mean (sd) | 36.1 (12.9) | | 31.4 (10.6) | | 9.73 | 2564 | < 0.001 |
|  | Median (iqr) | 33 (26-47) | | 29 (23-38) | |  |  |  |
|  | missing | 2 | | - | |  |  |  |
| IQ | |  |  |  |  |  |  |  |
|  | Mean (sd) | 103.4 (18.4) | | 84.9 (19.2) | | 19.74 | 1678 | < 0.001 |
|  | Median (iqr) | 103 (91-116) | | 83 (72-97) | |  |  |  |
|  | missing | 482 | | 406 | |  |  |  |

|  |  | Controls  n (%) | | Cases  n (%) | | χ² | df | p |
| --- | --- | --- | --- | --- | --- | --- | --- | --- |
| Sex | |  |  |  |  |  |  |  |
|  | Men | 706 | (47.2) | 657 | (61.3) | 50.4 | 1 | < 0.001 |
|  | Women | 791 | (52.8) | 414 | (38.7) |  |  |  |
|  | missing | - | | - | |  |  |  |
| Ethnicity | |  |  |  |  |  |  |  |
|  | White | 1,178 | (78.7) | 696 | (65.0) | 68.8 | 5 | < 0.001 |
|  | Black | 121 | (8.1) | 163 | (15.2) |  |  |  |
|  | Mixed heritage | 116 | (7.8) | 105 | (9.8) |  |  |  |
|  | Asian | 33 | (2.2) | 32 | (3.0) |  |  |  |
|  | North African | 24 | (1.6) | 43 | (4.0) |  |  |  |
|  | Other | 24 | (1.6) | 32 | (3.0) |  |  |  |
|  | missing | 1 | | - | |  |  |  |
| Education, highest level | |  |  |  |  |  |  |  |
|  | School, no qualifications | 72 | (4.8) | 168 | (15.9) | 238.2 | 5 | < 0.001 |
|  | School, qualifications | 197 | (13.2) | 277 | (26.1) |  |  |  |
|  | Tertiary | 431 | (28.9) | 252 | (23.8) |  |  |  |
|  | Vocational | 238 | (16.0) | 189 | (17.8) |  |  |  |
|  | University, undergraduate | 343 | (23.0) | 122 | (11.5) |  |  |  |
|  | University, postgraduate | 209 | (14.0) | 52 | (4.9) |  |  |  |
|  | missing | 7 | | 11 | |  |  |  |
| Economic status, 1 yr pre-assess. | |  |  |  |  |  |  |  |
|  | Employed | 892 | (65.9) | 569 | (54.6) | 74.5 | 2 | < 0.001 |
|  | Unemployed | 110 | (8.1) | 208 | (19.9) |  |  |  |
|  | Economically inact. (inc. stud.) | 352 | (26.0) | 266 | (25.5) |  |  |  |
|  | missing | 143 | | 28 | |  |  |  |
| Ever employed | |  |  |  |  |  |  |  |
|  | No | 92 | (6.2) | 118 | (11.0) | 19.8 | 1 | < 0.001 |
|  | Yes | 1,403 | (93.8) | 951 | (89.0) |  |  |  |
|  | missing | 2 | | 2 | |  |  |  |
| Long-term relationship | |  |  |  |  |  |  |  |
|  | No | 162 | (10.8) | 336 | (31.7) | 172.0 | 1 | < 0.001 |
|  | Yes | 1,333 | (89.2) | 724 | (68.3) |  |  |  |
|  | missing | 2 | | 11 | |  |  |  |
| Parental social class at birth | |  |  |  |  |  |  |  |
|  | Salariat | 415 | (29.2) | 228 | (24.3) | 24.8 | 3 | < 0.001 |
|  | Intermediate | 338 | (23.8) | 220 | (23.5) |  |  |  |
|  | Woking class | 659 | (46.4) | 461 | (49.2) |  |  |  |
|  | Long-term unemployed | 9 | (0.6) | 28 | (3.0) |  |  |  |
|  | missing | 76 | | 134 | |  |  |  |
| Long-term relationship | |  |  |  |  |  |  |  |
|  | No | 162 | (10.8) | 336 | (31.7) | 172.0 | 1 | < 0.001 |
|  | Yes | 1,333 | (89.2) | 724 | (68.3) |  |  |  |
|  | missing | 2 | | 11 | |  |  |  |
| Parental history of psychosis | |  |  |  |  |  |  |  |
|  | No | 1,309 | (98.3) | 864 | (92.7) | 44.1 | 1 | < 0.001 |
|  | Yes | 23 | (1.7) | 68 | (7.7) |  |  |  |
|  | missing | 165 | | 139 | |  |  |  |
| Diagnosis | |  |  |  |  |  |  |  |
|  | Schizophrenia | - | - | 527 | (49.2) | - | - | - |
|  | Other non-affective† | - | - | 244 | (22.4) | - | - | - |
|  | Affective | - | - | 304 | (28.4) | - | - | - |
|  | missing |  |  | - | |  |  |  |

† Includes schizoaffective disorder, delusional disorder, and psychosis NOS (which includes 26 with insufficient information to derive an OPCRIT diagnosis)

**Supplementary Table 2b.** Adversities (preset vs. absent) by case-control status.

|  | | Controls  n = 1,497  n (%) | | Cases  n = 1,071  n (%) | | χ² | df | p |
| --- | --- | --- | --- | --- | --- | --- | --- | --- |
| Parent died | |  |  |  |  |  |  |  |
|  | no | 1,373 | (93.0) | 938 | (89.2) | 10.8 | 1 | 0.001 |
|  | yes | 104 | (7.0) | 113 | (10.8) |  |  |  |
|  | missing | 20 | | 20 | |  |  |  |
| Separated, mother | |  |  |  |  |  |  |  |
|  | no | 1,368 | (92.2) | 889 | (84.3) | 39.2 | 1 | < 0.001 |
|  | yes | 115 | (7.8) | 165 | (15.7) |  |  |  |
|  | missing | 10 | | 17 | |  |  |  |
| Separated, father | |  |  |  |  |  |  |  |
|  | no | 1,227 | (83.1) | 757 | (71.9) | 45.4 | 1 | < 0.001 |
|  | yes | 250 | (16.9) | 296 | (28.1) |  |  |  |
|  | missing | 20 | | 18 | |  |  |  |
| Change school | |  |  |  |  |  |  |  |
|  | no | 913 | (61.3) | 563 | (53.6) | 14.8 | 1 | < 0.001 |
|  | yes | 577 | (38.7) | 487 | (46.4) |  |  |  |
|  | missing | 7 | | 21 | |  |  |  |
| Excluded | |  |  |  |  |  |  |  |
|  | no | 1,416 | (95.1) | 935 | (89.0) | 32.9 | 1 | < 0.001 |
|  | yes | 73 | (4.9) | 115 | (11.0) |  |  |  |
|  | missing | 8 | | 21 | |  |  |  |
| In care | |  |  |  |  |  |  |  |
|  | no | 1,469 | (98.6) | 994 | (94.5) | 34.5 | 1 | < 0.001 |
|  | yes | 21 | (1.4) | 58 | (5.5) |  |  |  |
|  | missing | 7 | | 19 | |  |  |  |
| Money problems | |  |  |  |  |  |  |  |
|  | no | 1,093 | (73.4) | 679 | (65.0) | 20.5 | 1 | < 0.001 |
|  | yes | 397 | (26.6) | 366 | (35.0) |  |  |  |
|  | missing | 7 | | 26 | |  |  |  |
| Neglect | |  |  |  |  |  |  |  |
|  | no | 1,407 | (94.4) | 920 | (87.7) | 35.6 | 1 | < 0.001 |
|  | yes | 84 | (5.6) | 129 | (12.3) |  |  |  |
|  | missing | 6 | | 22 | |  |  |  |
| Household Discord | |  |  |  |  |  |  |  |
|  | no | 926 | (62.1) | 537 | (51.5) | 28.1 | 1 | < 0.001 |
|  | yes | 566 | (37.9) | 506 | (48.5) |  |  |  |
|  | missing | 5 | | 28 | |  |  |  |
| Psychological Abuse | |  |  |  |  |  |  |  |
|  | no | 1,361 | (91.3) | 858 | (82.3) | 45.1 | 1 | < 0.001 |
|  | yes | 130 | (8.7) | 184 | (17.7) |  |  |  |
|  | missing | 6 | | 29 | |  |  |  |
| Physical Abuse | |  |  |  |  |  |  |  |
|  | no | 1,177 | (78.8) | 743 | (71.0) | 20.7 | 1 | < 0.001 |
|  | yes | 316 | (21.2) | 304 | (29.0) |  |  |  |
|  | missing | 4 | | 24 | |  |  |  |
| Sexual Abuse | |  |  |  |  |  |  |  |
|  | no | 1,377 | (92.2) | 912 | (87.5) | 15.5 | 1 | < 0.001 |
|  | yes | 116 | (7.8) | 130 | (12.5) |  |  |  |
|  | missing | 4 | | 29 | |  |  |  |
| Bullied | |  |  |  |  |  |  |  |
|  | no | 1,045 | (71.5) | 594 | (58.4) | 45.7 | 1 | < 0.001 |
|  | yes | 417 | (28.5) | 423 | (41.6) |  |  |  |
|  | missing | 35 | | 54 | |  |  |  |
| 1+ adversity | |  |  |  |  |  |  |  |
|  | no | 277 | (19.3) | 114 | (11.6) | 25.7 | 1 | < 0.001 |
|  | yes | 1,156 | (80.7) | 869 | (88.4) |  |  |  |
|  | missing | 64 | | 88 | |  |  |  |
| Lonely | |  |  |  |  |  |  |  |
|  | no | 1,185 | (79.6) | 670 | (64.4) | 72.5 | 1 | < 0.001 |
|  | yes | 303 | (20.4) | 370 | (35.6) |  |  |  |
|  | missing | 9 | | 31 | |  |  |  |
| Peer confidant | |  |  |  |  |  |  |  |
|  | no | 294 | (19.7) | 363 | (34.8) | 72.6 | 1 | < 0.001 |
|  | yes | 1,196 | (80.3) | 680 | (65.2) |  |  |  |
|  | missing | 7 | | 28 | |  |  |  |
| Adult confidant | |  |  |  |  |  |  |  |
|  | no | 411 | (27.6) | 431 | (41.3) | 51.9 | 1 | < 0.001 |
|  | yes | 1,079 | (72.4) | 613 | (58.7) |  |  |  |
|  | missing | 7 | | 27 | |  |  |  |

**Supplementary Table 3.** Main effects, each adversity (present vs. absent).

| Adversity | Controls  n = 1,497  n (%) | | Cases  n = 1,071  n (%) | | OR (1) | 95% CI | p | OR (2) | 95% CI | p | OR (3) | 95% CI | p |
| --- | --- | --- | --- | --- | --- | --- | --- | --- | --- | --- | --- | --- | --- |
| Parent died | 104 | (7.0) | 113 | (10.8) | 1.62 | 1.22-2.15 | 0.001 | 1.68 | 1.24-2.26 | 0.001 | 1.26 | 0.87-1.82 | 0.213 |
| Separated, mother | 115 | (7.8) | 165 | (15.7) | 2.23 | 1.73-2.89 | < 0.001 | 2.06 | 1.56-2.71 | < 0.001 | 1.55 | 1.12-2.13 | 0.008 |
| Separated, father | 250 | (16.9) | 296 | (28.1) | 1.92 | 1.58-2.34 | < 0.001 | 1.63 | 1.32-2.01 | < 0.001 | 1.36 | 1.06-1.74 | 0.016 |
| Change school | 577 | (38.7) | 487 | (46.4) | 1.40 | 1.19-1.65 | < 0.001 | 1.34 | 1.13-1.59 | 0.001 | 1.23 | 1.01-1.50 | 0.042 |
| Excluded | 73 | (4.9) | 115 | (11.0) | 2.33 | 1.71-3.16 | < 0.001 | 1.77 | 1.28-2.44 | 0.001 | 1.18 | 0.80-1.74 | 0.390 |
| In care | 21 | (1.4) | 58 | (5.5) | 4.33 | 2.58-7.26 | < 0.001 | 4.10 | 2.37-7.09 | < 0.001 | 2.17 | 1.16-4.06 | 0.016 |
| Money problems | 397 | (26.6) | 366 | (35.0) | 1.66 | 1.39-1.98 | < 0.001 | 1.65 | 1.37-2.00 | < 0.001 | 1.43 | 1.15-1.78 | 0.001 |
| Neglect | 84 | (5.6) | 129 | (12.3) | 2.69 | 2.00-3.62 | < 0.001 | 2.55 | 1.86-3.49 | < 0.001 | 1.92 | 1.28-2.89 | 0.002 |
| Household Discord | 566 | (37.9) | 506 | (48.5) | 1.57 | 1.33-1.85 | < 0.001 | 1.58 | 1.33-1.88 | < 0.001 | 1.61 | 1.31-1.97 | < 0.001 |
| Psychological Abuse | 130 | (8.7) | 184 | (17.7) | 2.46 | 1.93-3.14 | < 0.001 | 2.59 | 2.01-3.34 | < 0.001 | 2.27 | 1.68-3.06 | <0.001 |
| Physical Abuse | 316 | (21.2) | 304 | (29.0) | 1.65 | 1.36-2.00 | < 0.001 | 1.46 | 1.19-1.79 | < 0.001 | 1.21 | 0.95-1.54 | 0.131 |
| Sexual Abuse | 116 | (7.8) | 130 | (12.5) | 1.92 | 1.47-2.51 | < 0.001 | 2.23 | 1.66-2.97 | < 0.001 | 1.89 | 1.35-2.64 | < 0.001 |
| Bullied | 417 | (28.5) | 423 | (41.6) | 1.91 | 1.60-2.29 | < 0.001 | 1.85 | 1.53-2.23 | < 0.001 | 1.96 | 1.58-2.44 | < 0.001 |
| **1+ adversity** | **1,156** | **(80.7)** | **869** | **(88.4)** | **2.01** | **1.58-2.55** | **< 0.001** | **1.86** | **1.45-2.38** | **< 0.001** | **1.61** | **1.22-2.13** | **0.001** |
| Lonely | 303 | (20.4) | 370 | (35.6) | 2.29 | 1.90-2.75 | < 0.001 | 2.36 | 1.95-2.86 | < 0.001 | 2.37 | 1.88-3.00 | < 0.001 |
| Peer confidant | 1,196 | (80.3) | 680 | (65.2) | 0.46 | 0.39-01.56 | < 0.001 | 0.45 | 0.37-0.55 | < 0.001 | 0.55 | 0.44-0.70 | < 0.001 |
| Adult confidant | 1,079 | (72.4) | 613 | (58.7) | 0.53 | 0.45-0.63 | < 0.001 | 0.49 | 0.41-0.59 | < 0.001 | 0.56 | 0.45-0.69 | < 0.001 |

OR (1) Unadjusted; OR (2) Adjusted for age, sex, ethnicity; OR (3) Adjusted for age, sex, ethnicity, IQ, parental history of psychosis, parental social class at birth.

**Note:** Reported n and % are actual values; ORs, Cis, and p-values are estimated using imputed data.

**Supplementary Table 4.** Associations between each childhood adversity.

(a) Cases

|  | Parent died | Sep mother | Sep father | Change sch’l | Excluded | In care | Money prob | Neglect | H discord | Psych abuse | Phys abuse | Sex abuse | Bullied |
| --- | --- | --- | --- | --- | --- | --- | --- | --- | --- | --- | --- | --- | --- |
| Parent died | - |  |  |  |  |  |  |  |  |  |  |  |  |
| Sep mother | x^2^ 3.8  p 0.05 | - |  |  |  |  |  |  |  |  |  |  |  |
| Sep father | x^2^ 0.25  p 0.62 | x^2^ 227.2  p <0.01 | - |  |  |  |  |  |  |  |  |  |  |
| Change sch’l | x^2^ 0.1  p 0.98 | x^2^ 15.1  p <0.01 | x^2^ 25.5  p <0.01 | - |  |  |  |  |  |  |  |  |  |
| Excluded | x^2^ 3.3  p 0.07 | x^2^ 5.4  p 0.02 | x^2^ 12.1  p <0.01 | x^2^ 23.6  p <0.01 | - |  |  |  |  |  |  |  |  |
| In care | x^2^ 19.3  p <0.01 | x^2^ 116.4  p <0.01 | x^2^ 83.4  p <0.01 | x^2^ 16.1  p <0.01 | x^2^ 15.3  p <0.01 | - |  |  |  |  |  |  |  |
| Money prob | x^2^ 1.5  p 0.22 | x^2^ 13.2  p <0.01 | x^2^ 26.2  p <0.01 | x^2^ 8.2  p <0.01 | x^2^ 7.2  p <0.01 | x^2^ 5.0  p 0.03 | - |  |  |  |  |  |  |
| Neglect | x^2^ 12.2  p <0.01 | x^2^ 34.5  p <0.01 | x^2^ 12.8  p <0.01 | x^2^ 2.44  p 0.12 | x^2^ 8.7  p <0.01 | x^2^ 25.9  p <0.01 | x^2^ 160.5  p <0.01 | - |  |  |  |  |  |
| H Discord | x^2^ 7.2  p 0.01 * | x^2^ 5.8  p 0.02 | x^2^ 16.5  p <0.01 | x^2^ 6.5  p 0.01 | x^2^ 2.6  p 0.10 | x^2^ 4.2  p 0.04 | x^2^ 35.2  p <0.01 | x^2^ 26.4  p <0.01 | - |  |  |  |  |
| Psych abuse | x^2^ 0.1  p 0.73 | x^2^ 30.8  p <0.01 | x^2^ 20.3  p <0.01 | x^2^ 8.9  p <0.01 | x^2^ 5.5  0.02 | x^2^ 34.0  p <0.01 | x^2^ 16.4  p <0.01 | x^2^ 67.2  p <0.01 | x^2^ 60.3  p <0.01 | - |  |  |  |
| Phys abuse | x^2^ 6.6  p 0.01 | x^2^ 40.5  p <0.01 | x^2^ 36.3  p <0.01 | x^2^ 5.1  p 0.02 | x^2^ 5.1  p 0.02 | x^2^ 32.3  p <0.01 | x^2^ 34.1  p <0.01 | x^2^ 53.7  p <0.01 | x^2^ 48.1  p <0.01 | x^2^ 172.6  p <0.01 | - |  |  |
| Sex abuse | x^2^ 3.8  0.05 | x^2^ 35.8  p <0.01 | x^2^ 16.7  p <0.01 | x^2^ 4.6  p 0.03 | x^2^ 2.7  p 0.10 | x^2^ 17.8  p <0.01 | x^2^ 16.5  p <0.01 | x^2^ 16.8  p <0.01 | x^2^ 10.7  p <0.01 | x^2^ 77.1  p <0.01 | x^2^ 50.6  p <0.01 | - |  |
| Bullied | x^2^ 0.5  p 0.49 | x^2^ 0.12  p 0.73 | x^2^ 1.81  p 0.18 | x^2^ 5.9  p0.02 | x^2^ 0.3  p 0.60 | x^2^ 6.2  p 0.01 | x^2^ 0.6  p 0.45 | x^2^ 8.3  p <0.01 | x^2^ 32.4  p <0.01 | x^2^ 58.3  p <0.01 | x^2^ 21.6  p <0.01 | x^2^ 7.7  p 0.01 | - |

**Note 1:** 1 degree of freedom for all chi-square tests

**Note 2:** analyses were conducted using non-imputed data

* Household discord was less common among those who reported a parent had died

(b) Controls

|  | Parent died | Sep mother | Sep father | Change sch’l | Excluded | In care | Money prob | Neglect | H discord | Psych abuse | Phys abuse | Sex abuse | Bullied |
| --- | --- | --- | --- | --- | --- | --- | --- | --- | --- | --- | --- | --- | --- |
| Parent died | - |  |  |  |  |  |  |  |  |  |  |  |  |
| Sep mother | x^2^ 0.1  p 0.99 | - |  |  |  |  |  |  |  |  |  |  |  |
| Sep father | x^2^ 0.7  p 0.42 | x^2^ 315.0  p <0.01 | - |  |  |  |  |  |  |  |  |  |  |
| Change sch’l | x^2^ 0.1  p 0.94 | x^2^ 30.0  p <0.01 | x^2^ 30.9  p <0.01 | - |  |  |  |  |  |  |  |  |  |
| Excluded | x^2^ 0.7  p 0.39 | x^2^ 0.1  p 0.89 | x^2^ 2.2  p 0.14 | x^2^ 26.2  p <0.01 | - |  |  |  |  |  |  |  |  |
| In care | x^2^ 15.0  p <0.01 | x^2^ 72.5  p <0.01 | x^2^ 30.6  p <0.01 | x^2^ 3.0  p 0.08 | x^2^ 16.3  p <0.01 | - |  |  |  |  |  |  |  |
| Money prob | x^2^ 8.5  p <0.01 | x^2^ 5.2  p <0.01 | x^2^ 13.0  p <0.01 | x^2^ 6.5  p 0.01 | x^2^ 3.2  p 0.07 | x^2^ 8.3  p <0.01 | - |  |  |  |  |  |  |
| Neglect | x^2^ 13.3  p <0.01 | x^2^ 23.8  p <0.01 | x^2^ 21.0  p <0.01 | x^2^ 8.3  p <0.01 | x^2^ 12.8  p <0.01 | x^2^ 21.1  p <0.01 | x^2^ 117.2  p <0.01 | - |  |  |  |  |  |
| H Discord | x^2^ 10.2  p <0.01 | x^2^ 27.1  p <0.01 | x^2^ 30.9  p <0.01 | x^2^ 0.2  p 0.63 | x^2^ 1.2  p 0.28 | x^2^ 1.3  p 0.26 | x^2^ 42.2  p <0.01 | x^2^ 13.1  p <0.01 | - |  |  |  |  |
| Psych abuse | x^2^ 5.0  p 0.03 | x^2^ 20.7  p <0.01 | x^2^ 20.4  p <0.01 | x^2^ 1.6  p 0.20 | x^2^ 0.5  p 0.47 | x^2^ 11.6  p <0.01 | x^2^ 25.8  p <0.01 | x^2^ 57.4  p <0.01 | x^2^ 74.0  p <0.01 | - |  |  |  |
| Phys abuse | x^2^ 4.9  p 0.03 | x^2^ 27.6  p <0.01 | x^2^ 34.3  p <0.01 | x^2^ 6.6  p 0.01 | x^2^ 4.0  p 0.05 | x^2^ 7.9  p <0.01 | x^2^ 16.0  p <0.01 | x^2^ 55.7  p <0.01 | x^2^ 74.0  p <0.01 | x^2^ 122.2  p <0.01 | - |  |  |
| Sex abuse | x^2^ 0.2  p 0.69 | x^2^ 30.4  p <0.01 | x^2^ 41.5  p <0.01 | x^2^ 0.2  p 0.69 | x^2^ 0.1  p 0.86 | x^2^ 8.3  p <0.01 | x^2^ 6.9  p <0.01 | x^2^ 12.9  p <0.01 | x^2^ 26.0  p <0.01 | x^2^ 24.4  p <0.01 | x^2^ 26.7  p <0.01 | - |  |
| Bullied | x^2^ 1.1  p 0.30 | x^2^ 0.2  p 0.66 | x^2^ 3.2  p 0.08 | x^2^ 5.2  p 0.02 | x^2^ 0.1  p 0.75 | x^2^ 1.0  p 0.33 | x^2^ 9.0  p <0.01 | x^2^ 1.8  p 0.18 | x^2^ 29.1  p <0.01 | x^2^ 24.3  p <0.01 | x^2^ 24.1  p <0.01 | x^2^ 27.1  p <0.01 | - |

**Note 1:** 1 degree of freedom for all chi-square tests

**Note 2:** analyses were conducted using non-imputed data

**Supplementary Table 5.** Number of adversities, index.

| Adversity | Controls  n = 1,497  n (%) | | Cases  n = 1,071  n (%) | | OR (1) | 95% CI | p | OR (2) | 95% CI | p | OR (3) | 95% CI | p |
| --- | --- | --- | --- | --- | --- | --- | --- | --- | --- | --- | --- | --- | --- |
| 0 | 277 | (19.3) | 114 | (11.6) | 1.00 | - | - | 1.00 | - | - | 1.00 | - | - |
| 1 | 364 | (25.4) | 166 | (16.9) | 1.17 | 0.88-1.56 | 0.288 | 1.11 | 0.82-1.47 | 0.509 | 1.03 | 0.74-1.44 | 0.868 |
| 2 | 309 | (21.6) | 187 | (19.0) | 1.71 | 1.28-2.28 | < 0.001 | 1.67 | 1.24-2.25 | 0.001 | 1.52 | 1.09-2.12 | 0.014 |
| 3 | 228 | (15.9) | 163 | (16.6) | 2.10 | 1.55-2.84 | < 0.001 | 1.99 | 1.45-2.73 | < 0.001 | 1.75 | 1.23-2.50 | 0.002 |
| 4 | 126 | (8.8) | 116 | (11.8) | 2.64 | 1.88-3.71 | < 0.001 | 2.47 | 1.73-3.51 | < 0.001 | 1.99 | 1.34-2.95 | 0.001 |
| 5 | 64 | (4.5) | 95 | (9.6) | 4.14 | 2.80-6.13 | < 0.001 | 3.62 | 2.40-5.46 | < 0.001 | 2.79 | 1.73-4.49 | < 0.001 |
| 6 or more | 65 | (4.5) | 143 | (14.6) | 6.53 | 4.56-9.35 | < 0.001 | 6.02 | 4.13-8.78 | < 0.001 | 3.91 | 2.54-6.01 | < 0.001 |
| **3 + (vs. 0 - 2)** | **483** | **(33.7)** | **529** | **(52.7)** | **2.30** | **1.93-2.73** | **< 0.001** | **2.16** | **1.79-2.59** | **< 0.001** | **1.81** | **1.46-2.24** | **< 0.001** |
| **Score:** median (IQR) | **2** | **(1-3)** | **3** | **(1-4)** | **1.36** | **1.29-1.42** | **< 0.001** | **1.34** | **1.27-1.40** | **< 0.001** | **1.25** | **1.18-1.33** | **< 0.001** |

OR (1) Unadjusted; OR (2) Adjusted for age, sex, and ethnicity; OR (3) Adjusted for age, sex, ethnicity, IQ, parental history of psychosis, and parental social class at birth.

**Note (1):** Number of adversities entered as a continuous variable (0 to 6+): fully Adj. OR 1.25 (95% CI 1.18-1.33); for each additional adversity, odds of psychotic disorder increase by, on average, around 25%.

**Note (2):** Reported n and % are actual values; ORs, Cis, and p-values are estimated using imputed data.

**Supplementary Table 6.** Main effects, each adversity (present vs. absent) by sex.

| Adversity | | OR (1) | 95% CI | p | p int.* | OR (2) | 95% CI | p | p int.* | OR (3) | 95% CI | p | p int.* |
| --- | --- | --- | --- | --- | --- | --- | --- | --- | --- | --- | --- | --- | --- |
| Parent died | |  |  |  |  |  |  |  |  |  |  |  |  |
|  | men | 1.62 | 1.09-2.41 | 0.017 | 0.903 | 1.56 | 1.03-2.35 | 0.035 | 0.607 | 1.24 | 0.75-2.05 | 0.406 | 0.915 |
|  | women | 1.68 | 1.12-2.53 | 0.013 |  | 1.82 | 1.19-2.78 | 0.006 |  | 1.29 | 0.77-2.14 | 0.332 |  |
| Separated, mother | |  |  |  |  |  |  |  |  |  |  |  |  |
|  | men | 2.14 | 1.48-3.09 | <0.001 | 0.530 | 1.82 | 1.25-2.69 | 0.002 | 0.406 | 1.36 | 0.87-2.11 | 0.178 | 0.407 |
|  | women | 2.52 | 1.76-3.62 | <0.001 |  | 2.29 | 1.57-3.34 | <0.001 |  | 1.74 | 1.13-2.69 | 0.012 |  |
| Separated, father | |  |  |  |  |  |  |  |  |  |  |  |  |
|  | men | 1.96 | 1.49-2.58 | <0.001 | 0.924 | 1.59 | 1.19-2.11 | 0.001 | 0.770 | 1.33 | 0.96-1.85 | 0.085 | 0.885 |
|  | women | 2.00 | 1.50-2.66 | <0.001 |  | 1.68 | 1.25-2.26 | <0.001 |  | 1.38 | 0.97-1.96 | 0.072 |  |
| Change school | |  |  |  |  |  |  |  |  |  |  |  |  |
|  | men | 1.39 | 1.12-1.73 | 0.003 | 0.989 | 1.32 | 1.06-1.67 | 0.014 | 0.931 | 1.18 | 0.91-1.54 | 0.242 | 0.660 |
|  | women | 1.39 | 1.08-1.78 | 0.009 |  | 1.35 | 1.04-1.74 | 0.022 |  | 1.29 | 0.96-1.73 | 0.089 |  |
| Excluded | |  |  |  |  |  |  |  |  |  |  |  |  |
|  | men | 2.06 | 1.42-2.98 | <0.001 | 0.857 | 1.70 | 1.17-2.50 | 0.006 | 0.735 | 1.15 | 0.74-1.81 | 0.535 | 0.815 |
|  | women | 2.19 | 1.23-3.90 | 0.008 |  | 1.93 | 1.06-3.52 | 0.032 |  | 1.27 | 0.62-2.59 | 0.506 |  |
| In care | |  |  |  |  |  |  |  |  |  |  |  |  |
|  | men | 5.79 | 2.52-13.29 | <0.001 | 0.444 | 5.42 | 2.28-12.86 | <0.001 | 0.397 | 2.44 | 0.97-6.19 | 0.058 | 0.727 |
|  | women | 3.82 | 1.94-7.53 | <0.001 |  | 3.36 | 1.65-6.84 | 0.001 |  | 1.97 | 0.86-4.50 | 0.106 |  |
| Money problems | |  |  |  |  |  |  |  |  |  |  |  |  |
|  | men | 1.71 | 1.35-2.18 | <0.001 | 0.716 | 1.71 | 1.33-2.20 | <0.001 | 0.688 | 1.46 | 1.08-1.97 | 0.013 | 0.830 |
|  | women | 1.60 | 1.23-2.08 | <0.001 |  | 1.59 | 1.21-2.09 | 0.001 |  | 1.40 | 1.02-1.91 | 0.036 |  |
| Neglect | |  |  |  |  |  |  |  |  |  |  |  |  |
|  | men | 4.55 | 2.83-7.34 | <0.001 | 0.007 | 4.07 | 2.49-6.66 | <0.001 | 0.010 | 2.93 | 1.60-5.38 | <0.001 | 0.054 |
|  | women | 1.93 | 1.29-2.90 | 0.001 |  | 1.76 | 1.15-2.68 | 0.009 |  | 1.41 | 0.84-2.36 | 0.189 |  |
| Household Discord | |  |  |  |  |  |  |  |  |  |  |  |  |
|  | men | 1.66 | 1.33-2.08 | <0.001 | 0.782 | 1.58 | 1.26-1.99 | <0.001 | 0.965 | 1.68 | 1.29-2.20 | <0.001 | 0.610 |
|  | women | 1.59 | 1.24-2.04 | <0.001 |  | 1.57 | 1.22-2.03 | <0.001 |  | 1.52 | 1.14-2.03 | 0.005 |  |
| Psychological Abuse | |  |  |  |  |  |  |  |  |  |  |  |  |
|  | men | 2.72 | 1.88-3.93 | <0.001 | 0.868 | 2.63 | 1.80-3.83 | <0.001 | 0.928 | 2.31 | 1.49-3.60 | <0.001 | 0.898 |
|  | women | 2.61 | 1.89-3.62 | <0.001 |  | 2.57 | 1.84-3.59 | <0.001 |  | 2.23 | 1.51-3.29 | <0.001 |  |
| Physical Abuse | |  |  |  |  |  |  |  |  |  |  |  |  |
|  | men | 1.35 | 1.04-1.74 | 0.022 | 0.016 | 1.20 | 0.92-1.57 | 0.169 | 0.028 | 1.03 | 0.76-1.41 | 0.839 | 0.127 |
|  | women | 2.11 | 1.60-2.79 | <0.001 |  | 1.84 | 1.38-2.46 | <0.001 |  | 1.46 | 1.03-2.05 | 0.032 |  |
| Sexual Abuse | |  |  |  |  |  |  |  |  |  |  |  |  |
|  | men | 1.88 | 1.17-3.01 | 0.009 | 0.257 | 1.90 | 1.16-3.10 | 0.011 | 0.431 | 1.59 | 0.90-2.82 | 0.120 | 0.471 |
|  | women | 2.61 | 1.88-3.63 | <0.001 |  | 2.40 | 1.70-3.38 | <0.001 |  | 2.04 | 1.37-3.03 | <0.001 |  |
| Bullied | |  |  |  |  |  |  |  |  |  |  |  |  |
|  | men | 1.98 | 1.56-2.52 | <0.001 | 0.789 | 1.90 | 1.49-2.43 | <0.001 | 0.707 | 2.00 | 1.50-2.67 | <0.001 | 0.825 |
|  | women | 1.89 | 1.46-2.46 | <0.001 |  | 1.78 | 1.36-2.33 | <0.001 |  | 1.91 | 1.41-2.61 | <0.001 |  |
| **Any adversity** | |  |  |  |  |  |  |  |  |  |  |  |  |
|  | men | 2.00 | 1.46-2.75 | <0.001 | 0.993 | 1.87 | 1.35-2.59 | <0.001 | 0.941 | 1.66 | 1.15-2.39 | 0.007 | 0.816 |
|  | women | 2.01 | 1.39-2.90 | 0.001 |  | 1.84 | 1.26-2.67 | 0.002 |  | 1.55 | 1.03-2.35 | 0.037 |  |
| Lonely | |  |  |  |  |  |  |  |  |  |  |  |  |
|  | men | 2.27 | 1.76-2.94 | <0.001 | 0.509 | 2.19 | 1.68-2.86 | <0.001 | 0.435 | 2.15 | 1.58-2.94 | <0.001 | 0.366 |
|  | women | 2.57 | 1.97-3.36 | <0.001 |  | 2.55 | 1.94-3.35 | <0.001 |  | 2.63 | 1.90-3.64 | <0.001 |  |
| Adult confidant | |  |  |  |  |  |  |  |  |  |  |  |  |
|  | men | 0.50 | 0.40-0.63 | <0.001 | 0.544 | 0.48 | 0.38-0.61 | <0.001 | 0.733 | 0.56 | 0.42-0.74 | <0.001 | 0.980 |
|  | women | 0.56 | 0.43-0.73 | <0.001 |  | 0.51 | 0.39-0.67 | <0.001 |  | 0.56 | 0.41-0.76 | 0.001 |  |
| Peer confidant | |  |  |  |  |  |  |  |  |  |  |  |  |
|  | men | 0.49 | 0.39-0.63 | <0.001 | 0.856 | 0.45 | 0.35-0.58 | <0.001 | 0.930 | 0.57 | 0.42-0.76 | <0.001 | 0.792 |
|  | women | 0.48 | 0.36-0.64 | <0.001 |  | 0.46 | 0.34-0.62 | <0.001 |  | 0.54 | 0.38-0.76 | 0.001 |  |

OR (1) Unadjusted; OR (2) Adjusted for age, ethnicity; OR (3) Adjusted for age, ethnicity, IQ, parental history of psychosis, parental social class at birth.

* p value for interaction term

**Supplementary Table 7.** Main effects for each type of childhood adversity, by diagnosis.

|  | | Controls  n = 1,497 | | Cases, Non-Affective  n = 767 | | Cases, Affective  n = 304 | |
| --- | --- | --- | --- | --- | --- | --- | --- |
| Parent died | |  |  |  |  |  |  |
|  | Yes (n* (%)) | 104 | (7.0) | 89 | (11.8) | 24 | (8.1) |
|  | OR (1) (95% CI) | 1.00 (-) | | 1.90 (1.40-2.58) | | 1.08 (0.68-1.71) | |
|  | OR (2) (95% CI) | 1.00 (-) | | 2.01 (1.45-2.80) | | 1.09 (0.67-1.76) | |
|  | OR (3) (95% CI) | 1.00 (-) | | 1.54 (1.04-2.90) | | 0.83 (0.48-1.46) | |
| Sep, mother | |  |  |  |  |  |  |
|  | Yes (n* (%)) | 115 | (7.8) | 118 | 115 | (7.8) | 118 |
|  | OR (1) (95% CI) | 1.00 (-) | | 2.27 (1.72-3.01) | | 2.20 (1.52-3.18) | |
|  | OR (2) (95% CI) | 1.00 (-) | | 2.06 (1.51-2.80) | | 1.97 (1.34-2.89) | |
|  | OR (3) (95% CI) | 1.00 (-) | | 1.52 (1.06-2.19) | | 1.50 (0.96-2.32) | |
| Sep, father | |  |  |  |  |  |  |
|  | Yes (n* (%)) | 250 | (16.9) | 220 | 250 | (16.9) | 220 |
|  | OR (1) (95% CI) | 1.00 (-) | | 2.05 (1.65-2.54) | | 1.65 (1.23-2.23) | |
|  | OR (2) (95% CI) | 1.00 (-) | | 1.72 (1.36-2.17) | | 1.41 (1.03-1.92) | |
|  | OR (3) (95% CI) | 1.00 (-) | | 1.46 (1.11-1.92) | | 1.16 (0.82-1.65) | |
| Changed school | |  |  |  |  |  |  |
|  | Yes (n* (%)) | 577 | (38.7) | 342 | 577 | (38.7) | 342 |
|  | OR (1) (95% CI) | 1.00 (-) | | 1.41 (1.17-1.69) | | 1.43 (1.11-1.85) | |
|  | OR (2) (95% CI) | 1.00 (-) | | 1.31 (1.08-1.59) | | 1.42 (1.09-1.83) | |
|  | OR (3) (95% CI) | 1.00 (-) | | 1.19 (0.95-1.49) | | 1.34 (1.00-1.80) | |
| Excluded | |  |  |  |  |  |  |
|  | Yes (n* (%)) | 73 | (4.9) | 92 | 73 | (4.9) | 92 |
|  | OR (1) (95% CI) | 1.00 (-) | | 2.63 (1.89-3.64) | | 1.60 (0.97-2.62) | |
|  | OR (2) (95% CI) | 1.00 (-) | | 1.89 (1.32-2.66) | | 1.42 (0.86-2.36) | |
|  | OR (3) (95% CI) | 1.00 (-) | | 1.19 (0.78-1.82) | | 1.15 (0.65-2.02) | |
| In care | |  |  |  |  |  |  |
|  | Yes (n* (%)) | 21 | (1.4) | 48 | 21 | (1.4) | 48 |
|  | OR (1) (95% CI) | 1.00 (-) | | 5.09 (2.97-8.70) | | 2.58 (1.18-5.62) | |
|  | OR (2) (95% CI) | 1.00 (-) | | 4.86 (2.72-8.69) | | 2.50 (1.13-5.57) | |
|  | OR (3) (95% CI) | 1.00 (-) | | 2.50 (1.23-4.90) | | 1.37 (0.56-3.33) | |
| Money problems | |  |  |  |  |  |  |
|  | Yes (n* (%)) | 397 | (26.6) | 245 | 397 | (26.6) | 245 |
|  | OR (1) (95% CI) | 1.00 (-) | | 1.62 (1.33-1.98) | | 1.76 (1.35-2.30) | |
|  | OR (2) (95% CI) | 1.00 (-) | | 1.61 (1.30-1.99) | | 1.76 (1.34-2.31) | |
|  | OR (3) (95% CI) |  | |  | |  | |
| Neglect | |  |  |  |  |  |  |
|  | Yes (n* (%)) | 84 | (5.6) | 85 | (11.4) | 44 | (14.7) |
|  | OR (1) (95% CI) | 1.00 (-) | | 2.80 (2.01-3.90) | | 2.53 (1.69-3.78) | |
|  | OR (2) (95% CI) | 1.00 (-) | | 2.81 (1.96-4.03) | | 2.29 (1.50-3.48) | |
|  | OR (3) (95% CI) | 1.00 (-) | | 2.13 (1.35-3.34) | | 1.72 (1.04-2.84) | |
| Household discord | |  |  |  |  |  |  |
|  | Yes (n* (%)) | 566 | (37.9) | 350 | (47.1) | 156 | (52.0) |
|  | OR (1) (95% CI) | 1.00 (-) | | 1.50 (1.25-1.81) | | 1.81 (1.40-2.33) | |
|  | OR (2) (95% CI) | 1.00 (-) | | 1.52 (1.25-1.85) | | 1.77 (1.37-2.30) | |
|  | OR (3) (95% CI) | 1.00 (-) | | 1.55 (1.23-1.96) | | 1.87 (1.40-2.51) | |
| Psych abuse | |  |  |  |  |  |  |
|  | Yes (n* (%)) | 130 | (8.7) | 127 | (17.1) | 57 | (19.1) |
|  | OR (1) (95% CI) | 1.00 (-) | | 2.39 (1.83-3.12) | | 2.66 (1.89-3.76) | |
|  | OR (2) (95% CI) | 1.00 (-) | | 2.63 (1.98-3.50) | | 2.71 (1.91-3.85) | |
|  | OR (3) (95% CI) | 1.00 (-) | | 2.24 (1.60-3.14) | | 2.38 (1.58-3.57) | |
| Phys abuse | |  |  |  |  |  |  |
|  | Yes (n* (%)) | 316 | (21.2) | 223 | (29.9) | 81 | (27.0) |
|  | OR (1) (95% CI) | 1.00 (-) | | 1.84 (1.49-2.29) | | 1.29 (0.96-1.75) | |
|  | OR (2) (95% CI) | 1.00 (-) | | 1.61 (1.28-2.03) | | 1.14 (0.84-1.55) | |
|  | OR (3) (95% CI) | 1.00 (-) | | 1.37 (1.04-1.80) | | 0.94 (0.66-1.34) | |
| Sex abuse | |  |  |  |  |  |  |
|  | Yes (n* (%)) | 116 | (7.8) | 90 | (12.1) | 40 | (13.5) |
|  | OR (1) (95% CI) | 1.00 (-) | | 1.79 (1.33-2.40) | | 2.19 (1.50-3.21) | |
|  | OR (2) (95% CI) | 1.00 (-) | | 2.27 (1.65-3.13) | | 2.14 (1.43-3.20) | |
|  | OR (3) (95% CI) | 1.00 (-) | | 1.92 (1.32-2.79) | | 1.87 (1.18-2.94) | |
| Bullied | |  |  |  |  |  |  |
|  | Yes (n* (%)) | 417 | (28.5) | 296 | (40.6) | 127 | (44.1) |
|  | OR (1) (95% CI) | 1.00 (-) | | 1.78 (1.46-2.17) | | 2.28 (1.73-3.01) | |
|  | OR (2) (95% CI) | 1.00 (-) | | 1.73 (1.40-2.13) | | 2.15 (1.62-2.85) | |
|  | OR (3) (95% CI) | 1.00 (-) | | 1.82 (1.43-2.31) | | 2.30 (1.67-3.16) | |
| Any adversity | |  |  |  |  |  |  |
|  | Yes (n* (%)) | 1,156 | (80.7) | 616 | (87.4) | 253 | (91.0) |
|  | OR (1) (95% CI) | 1.00 (-) | | 1.90 (1.46-2.48) | | 2.42 (1.56-3.76) | |
|  | OR (2) (95% CI) | 1.00 (-) | | 1.73 (1.31-2.28) | | 2.31 (1.48-3.60) | |
|  | OR (3) (95% CI) | 1.00 (-) | | 1.49 (1.09-2.03) | | 1.92 (1.21-3.05) | |
| Lonely | |  |  |  |  |  |  |
|  | Yes (n* (%)) | 303 | (20.4) | 242 | (32.6) | 128 | (43.0) |
|  | OR (1) (95% CI) | 1.00 (-) | | 2.00 (1.63-2.45) | | 3.11 (2.37-4.08) | |
|  | OR (2) (95% CI) | 1.00 (-) | | 2.06 (1.66-2.56) | | 3.11 (2.36-4.10) | |
|  | OR (3) (95% CI) | 1.00 (-) | | 2.05 (1.58-2.65) | | 3.11 (2.26-4.30) | |
| Peer confidant | |  |  |  |  |  |  |
|  | Yes (n* (%)) | 1,196 | (80.3) | 465 | (62.5) | 215 | (71.9) |
|  | OR (1) (95% CI) | 1.00 (-) | | 0.42 (0.34-0.51) | | 0.62 (0.47-0.83) | |
|  | OR (2) (95% CI) | 1.00 (-) | | 0.41 (0.33-0.51) | | 0.58 (0.43-0.79) | |
|  | OR (3) (95% CI) | 1.00 (-) | | 0.50 (0.39-0.65) | | 0.68 (0.49-0.96) | |
| Adult confidant | |  |  |  |  |  |  |
|  | Yes (n* (%)) | 1,079 | (72.4) | 429 | (57.7) | 184 | (61.3) |
|  | OR (1) (95% CI) | 1.00 (-) | | 0.50 (0.41-.60) | | 0.63 (0.48-0.82) | |
|  | OR (2) (95% CI) | 1.00 (-) | | 0.46 (0.37-0.56) | | 0.61 (0.46-0.80) | |
|  | OR (3) (95% CI) | 1.00 (-) | | 0.53 (0.42-0.68) | | 0.65 (0.48-0.90) | |

OR (1) Unadjusted; OR (2) Adjusted for age, sex, ethnicity; OR (3) Adjusted for age, sex, ethnicity, IQ, parental history of psychosis, parental social class at birth.

**Supplementary Table 8a.** Adversity (any present vs. none; 3 plus; score) by site.

| Site | Any  Unadj. OR | 95% CI | p | 3 +  Unadj. OR | 95% CI | p | Score  Unadj. OR* | 95% CI | p |
| --- | --- | --- | --- | --- | --- | --- | --- | --- | --- |
| England: London | 2.23 | 0.79-6.27 | 0.129 | 2.07 | 1.35-3.17 | 0.001 | 1.44 | 1.28-1.62 | < 0.001 |
| England: Cambridge | 3.80 | 0.83-17.33 | 0.085 | 2.75 | 1.28-5.88 | 0.009 | 1.36 | 1.11-1.66 | < 0.001 |
| Holland: Amsterdam | 1.81 | 0.82-4.03 | 0.144 | 2.13 | 1.18-3.84 | 0.013 | 1.29 | 1.11-1.49 | 0.001 |
| Holland: Gouda & Voorhout | 2.04 | 0.96-4.34 | 0.064 | 2.65 | 1.49-4.69 | 0.001 | 1.39 | 1.17-1.64 | < 0.001 |
| France: Paris | 2.25 | 0.72-7.04 | 0.163 | 2.57 | 1.19-5.55 | 0.016 | 1.32 | 1.10-1.59 | 0.003 |
| France: Puy De Dome | - | - | - | 10.39 | 2.47-43.68 | 0.001 | 2.01 | 1.24-3.25 | 0.004 |
| Spain: Madrid | 12.07 | 2.52-57.68 | 0.002 | 1.70 | 0.64-4.55 | 0.287 | 1.42 | 1.08-1.86 | 0.012 |
| Spain: Barcelona | 2.48 | 0.60-10.34 | 0.212 | 0.76 | 0.28-2.07 | 0.597 | 1.13 | 0.86-1.48 | 0.377 |
| Spain: Oviedo | 5.47 | 1.62-18.51 | 0.006 | 1.64 | 0.61-4.41 | 0.328 | 1.37 | 1.07-1.75 | 0.012 |
| Spain: Valencia | 1.85 | 0.73-4.73 | 0.198 | 9.70 | 1.18-79.67 | 0.034 | 1.74 | 1.12-2.70 | 0.015 |
| Spain: Galicia | 3.43 | 0.98-11.96 | 0.053 | 2.95 | 0.97-8.94 | 0.056 | 1.48 | 1.07-2.04 | 0.017 |
| Spain: Cuenca | 0.42 | 0.12-1.42 | 0.161 | 3.59 | 0.71-18.26 | 0.124 | 1.14 | 0.75-1.73 | 0.532 |
| Italy: Bologna | 5.45 | 1.71-17.32 | 0.004 | 3.81 | 1.70-8.55 | 0.001 | 1.75 | 1.33-2.32 | < 0.001 |
| Italy: Palermo | 1.04 | 0.46-2.36 | 0.932 | 0.95 | 0.45-1.98 | 0.882 | 1.12 | 0.92-1.36 | 0.257 |
| Italy: Verona | 1.07 | 0.53-2.18 | 0.850 | 1.68 | 0.66-4.29 | 0.277 | 1.21 | 0.98-1.50 | 0.074 |
| Brazil: Ribeirao Preto | 1.54 | 0.83-2.88 | 0.170 | 2.85 | 1.95-4.15 | < 0.001 | 1.38 | 1.23-1.54 | < 0.001 |
| interaction by site | F 1.66, p 0.056 | | | F 1.44, p 0.119 | | | F 1.07, p 0.380 | | |

* Odds ratios express the estimated increase in odds of psychosis for each additional adversity

**Note:** Reported n and % are actual values; ORs, Cis, and p-values are estimated using imputed data.

**Supplementary Table 8b.** Moderate or Marked Adversity (any present vs. none; 3 plus; score) by site.

| Site | Any Mod*  Unadj. OR | 95% CI | p | 3 + Mod *  Unadj. OR* | 95% CI | p | Score Mod*  Unadj. OR | 95% CI | p |
| --- | --- | --- | --- | --- | --- | --- | --- | --- | --- |
| England: London | 2.06 | 1.32-3.21 | 0.001 | 3.90 | 2.04-7.47 | < 0.001 | 1.51 | 1.26-1.81 | <0.001 |
| England: Cambridge | 1.77 | 0.79-3.95 | 0.163 | 1.51 | 0.48-4.78 | 0.485 | 1.35 | 0.98-1.86 | 0.069 |
| Holland: Amsterdam | 1.14 | 0.62-2.07 | 0.675 | 1.41 | 0.53-3.79 | 0.491 | 1.06 | 0.83-1.36 | 0.646 |
| Holland: Gouda & Voorhout | 1.94 | 1.09-3.44 | 0.024 | 3.95 | 0.83-18.74 | 0.084 | 1.76 | 1.26-2.47 | 0.001 |
| France: Paris | 1.21 | 0.58-2.51 | 0.619 | 3.42 | 0.76-15.40 | 0.109 | 1.21 | 0.85-1.72 | 0.280 |
| France: Puy De Dome | 11.89 | 2.46-57.38 | 0.002 | 6.49 | 0.55-77.34 | 1.48 | 2.92 | 1.41-6.05 | 0.004 |
| Spain: Madrid | 2.49 | 0.95-6.56 | 0.065 | 2.12 | 0.36-12.34 | 0.403 | 1.42 | 0.90-2.24 | 0.129 |
| Spain: Barcelona | 3.75 | 1.31-10.67 | 0.013 | 1.27 | 0.29-5.57 | 0.752 | 1.68 | 1.06-2.68 | 0.028 |
| Spain: Oviedo | 4.73 | 1.73-12.86 | 0.002 | 2.01 | 0.32-12.62 | 0.457 | 1.75 | 1.04-2.94 | 0.037 |
| Spain: Valencia | 3.07 | 0.91-10.41 | 0.072 | ** | ** | ** | 1.83 | 0.86-3.87 | 0.114 |
| Spain: Galicia | 2.06 | 0.68-6.19 | 0.200 | 3.11 | 0.27-36.17 | 0.365 | 1.55 | 0.84-2.85 | 0.162 |
| Spain: Cuenca | 0.94 | 0.27-3.29 | 0.928 | ** | ** | ** | 1.63 | 0.74-3.57 | 0.225 |
| Italy: Bologna | 1.93 | 0.96-3.87 | 0.065 | ** | ** | ** | 1.73 | 1.06-2.82 | 0.027 |
| Italy: Palermo | 0.98 | 0.47-2.02 | 0.951 | 2.30 | 0.14-39.17 | 0.563 | 0.96 | 0.58-1.60 | 0.884 |
| Italy: Verona | 1.47 | 0.74-2.93 | 0.276 | 2.94 | 0.31-27.90 | 0.345 | 1.34 | 0.90-1.98 | 0.152 |
| Brazil: Ribeirao Preto | 1.85 | 1.28-2.67 | 0.001 | 9.74 | 3.30-28.74 | < 0.001 | 1.53 | 1.26-1.87 | <0.001 |
| interaction by site | F 1.38, p 0.145 | | | F 0.85, p 0.595 | | | F 1.17, p 0.287 | | |

* Odds ratios express the estimated increase in odds of psychosis for each additional adversity; ** No controls with 3 + moderate or marked adversities

**Note:** Reported n and % are actual values; ORs, Cis, and p-values are estimated using imputed data.

**Supplementary Table 9.** Incidence rates and prevalence of adversities in controls, by site.

|  | adj. incidence rate† | % of controls with 1 + any adversity(ies) | | % of controls with 3 + any adversity(ies) | | % of controls with 1 + moderate adversity(ies) | |
| --- | --- | --- | --- | --- | --- | --- | --- |
|  |  | unweighted | weighted* | unweighted | weighted* | unweighted | weighted* |
| UK: London | 45.8 | 93.8 | 93.4 | 54.9 | 54.7 | 58.5 | 57.6 |
| UK: Cambridge | 20.8 | 83.8 | 82.8 | 34.3 | 34.2 | 55.1 | 55.0 |
| Hollland: Amsterdam | 38.5 | 78.9 | 80.2 | 40.0 | 40.0 | 54.4 | 53.8 |
| Holland: Gouda & Voorhout | 21.8 | 77.6 | 77.0 | 31.8 | 33.2 | 50.5 | 52.0 |
| France: Paris (Val-de-Marne) | 41.5 | 81.7 | 81.1 | 37.8 | 36.7 | 48.2 | 47.5 |
| France: Puy de Dome | 21.0 | 73.9 | 72.3 | 26.1 | 22.5 | 27.3 | 22.5 |
| Spain: Madrid | 19.5 | 60.5 | 60.7 | 26.3 | 25.8 | 26.3 | 25.6 |
| Spain: Barcelona | 13.0 | 77.8 | 77.4 | 44.4 | 44.8 | 30.6 | 30.8 |
| Spain: Oviedo | 17.6 | 61.5 | 54.0 | 25.6 | 17.9 | 24.3 | 18.1 |
| Spain: Valencia | 16.3 | 54.8 | 57.2 | 3.2 | 5.6 | 12.5 | 11.9 |
| Spain: Cuenca | 13.9 | 79.0 | 75.1 | 7.9 | 9.0 | 29.0 | 31.5 |
| Spain: Santiago | 6.0 | 63.2 | 74.7 | 21.1 | 20.0 | 23.7 | 30.1 |
| Italy: Bologna | 23.2 | 75.4 | 75.0 | 18.0 | 25.2 | 44.4 | 38.1 |
| Italy: Palermo | 14.5 | 80.0 | 75.0 | 30.0 | 28.9 | 39.6 | 38.2 |
| Italy: Verona | 18.5 | 68.4 | 69.6 | 13.3 | 16.1 | 30.7 | 33.5 |
| Brazil: Ribeirao Preto | 24.5 | 88.0 | 87.7 | 36.1 | 34.9 | 41.5 | 40.8 |
| Spearman’s rho | - | 0.49 (p 0.058) | 0.55 (p 0.029) | 0.52 (p 0.040) | 0.56 (p 0.025) | 0.73 (p 0.002) | 0.64 (p 0.009) |
| Pearson’s r | - | 0.57 (p 0.020) | 0.53 (p 0.034) | 0.61 (p 0.012) | 0.64 (p 0.007) | 0.72 (p 0.002) | 0.65 (p 0.007) |

† adjusted for age, sex, ethnic group

* estimated % weigthed using post-stratification weights to account for known differences between control sample and population at risk by age, sex, and ethnic group

**Supplementary Table 10.** Childhood adversities and psychotic disorder, by frequency.

| Adversity | | Controls  n = 1,497  n* (%) | | Cases  n = 1,071  n* (%) | | OR (1) | 95% CI | p | OR (2) | 95% CI | p | OR (3) | 95% CI | p |
| --- | --- | --- | --- | --- | --- | --- | --- | --- | --- | --- | --- | --- | --- | --- |
| Household Discord | |  |  |  |  |  |  |  |  |  |  |  |  |  |
|  | None | 925 | (62.8) | 536 | (51.9) | 1.00 | - |  | 1.00 | - |  | 1.00 | - |  |
|  | Less than monthly | 136 | (9.2) | 100 | (9.7) | 1.22 | 0.91-1.63 | 0.191 | 1.20 | 0.89-1.63 | 0.241 | 1.14 | 0.80-1.62 | 0.465 |
|  | Monthly | 411 | (27.9) | 397 | (38.4) | 1.67 | 1.40-1.99 | <0.001 | 1.70 | 1.41-2.05 | <0.001 | 1.78 | 1.43-2.22 | <0.001 |
| Psychological Abuse | |  |  |  |  |  |  |  |  |  |  |  |  |  |
|  | None | 1,360 | (91.5) | 858 | (83.3) | 1.00 | - |  | 1.00 | - |  | 1.00 | - |  |
|  | Less than monthly | 37 | (2.5) | 53 | (5.2) | 2.31 | 1.46-3.64 | <0.001 | 2.30 | 1.44-3.69 | 0.001 | 1.79 | 1.03-3.11 | 0.038 |
|  | Monthly | 90 | (6.1) | 119 | (11.6) | 2.16 | 1.61-2.89 | <0.001 | 2.29 | 1.68-3.11 | <0.001 | 1.98 | 1.37-2.86 | <0.001 |
| Physical Abuse | |  |  |  |  |  |  |  |  |  |  |  |  |  |
|  | None | 1,176 | (79.2) | 743 | (71.9) | 1.00 | - |  | 1.00 | - |  | 1.00 | - |  |
|  | Less than monthly | 154 | (10.4) | 132 | (12.8) | 1.33 | 1.02-1.73 | 0.035 | 1.20 | 0.91-1.58 | 0.206 | 1.00 | 0.72-1.38 | 0.993 |
|  | Monthly | 155 | (10.4) | 158 | (15.3) | 1.64 | 1.28-2.10 | <0.001 | 1.40 | 1.08-1.82 | 0.012 | 1.13 | 0.83-1.55 | .438 |
| Sexual Abuse | |  |  |  |  |  |  |  |  |  |  |  |  |  |
|  | None | 1,376 | (92.6) | 912 | (88.5) | 1.00 | - |  | 1.00 | - |  | 1.00 | - |  |
|  | Less than monthly | 82 | (5.5) | 85 | (8.3) | 1.41 | 1.01-1.95 | 0.042 | 1.64 | 1.16-2.32 | 0.005 | 1.43 | 0.96-2.15 | 0.081 |
|  | Monthly | 28 | (1.9) | 33 | (3.2) | 1.73 | 1.04-2.90 | 0.036 | 1.97 | 1.15-3.39 | 0.014 | 1.84 | 0.98-3.47 | 0.058 |
| Bullying | |  |  |  |  |  |  |  |  |  |  |  |  |  |
|  | None | 1,045 | (71.5) | 594 | (58.5) | 1.00 | - |  | 1.00 | - |  | 1.00 | - |  |
|  | Less than monthly | 95 | (6.5) | 103 | (10.1) | 1.94 | 1.42-2.65 | <0.001 | 2.00 | 1.44-2.77 | <0.001 | 2.27 | 1.55-3.30 | <0.001 |
|  | Monthly | 321 | (22.0) | 319 | (31.4) | 1.77 | 1.45-2.15 | <0.001 | 1.68 | 1.39-2.06 | <0.001 | 1.74 | 1.37-2.21 | <0.001 |
| Any‡ | |  |  |  |  |  |  |  |  |  |  |  |  |  |
|  | None | 572 | (40.2) | 265 | (27.8) | 1.00 | - |  | 1.00 | - |  | 1.00 | - |  |
|  | Less than monthly | 195 | (13.7) | 128 | (13.4) | 1.49 | 1.14-1.94 | 0.004 | 1.48 | 1.12-1.95 | 0.006 | 1.39 | 1.01-1.93 | 0.045 |
|  | Monthly | 655 | (46.1) | 562 | (58.9) | 1.98 | 1.64-2.40 | <0.001 | 1.98 | 1.62-2.42 | <0.001 | 2.10 | 1.67-2.66 | <0.001 |

OR (1) Unadjusted

OR (2) Adjusted for age, sex, and ethnicity

OR (3) Adjusted for age, sex, ethnicity, IQ, parental history of psychosis, and parental social class at birth.

* n is number with complete data

‡ Any adversity, highest frequency

**Supplementary Table 11.** Childhood adversities and psychotic disorder, by age of first exposure.

| Any adversity | | Controls  n = 1,497  n* (%) | | Cases  n = 1,071  n* (%) | | OR (1) | 95% CI | p | OR (2) | 95% CI | p | OR (3) | 95% CI | p |
| --- | --- | --- | --- | --- | --- | --- | --- | --- | --- | --- | --- | --- | --- | --- |
| Household Discord | |  |  |  |  |  |  |  |  |  |  |  |  |  |
|  | None | 925 | (63.9) | 536 | (53.3) | 1.00 | - | - | 1.00 | - | - | 1.00 | - | - |
|  | 0 – 11 years | 384 | (26.5) | 371 | (36.9) | 1.68 | 1.40-2.01 | <0.001 | 1.73 | 1.44-2.10 | <0.001 | 1.72 | 1.38-2.15 | <0.001 |
|  | 12 – 16 years | 138 | (9.5) | 99 | (9.8) | 1.25 | 0.94-1.66 | 0.122 | 1.16 | 0.86-1.56 | 0.339 | 1.29 | 0.92-1.80 | 0.142 |
| Psychological Abuse | |  |  |  |  |  |  |  |  |  |  |  |  |  |
|  | None | 1,360 | (91.8) | 858 | (84.2) | 1.00 | - | - | 1.00 | - | - | 1.00 | - | - |
|  | 0 – 11 years | 86 | (5.8) | 125 | (12.3) | 2.66 | 2.01-3.51 | <0.001 | 2.91 | 2.17-3.89 | <0.001 | 2.38 | 1.68-3.35 | <0.001 |
|  | 12 – 16 years | 35 | (2.4) | 36 | (3.5) | 2.01 | 1.25-3.22 | 0.004 | 1.89 | 1.15-3.11 | 0.013 | 1.99 | 1.10-3.61 | 0.023 |
| Physical Abuse | |  |  |  |  |  |  |  |  |  |  |  |  |  |
|  | None | 1,176 | (79.6) | 743 | (72.9) | 1.00 | - | - | 1.00 | - | - | 1.00 | - | - |
|  | 0 – 11 years | 257 | (17.4) | 222 | (21.8) | 1.56 | 1.27-1.91 | <0.001 | 1.40 | 1.13-1.74 | 0.002 | 1.20 | 0.92-1.56 | 0.178 |
|  | 12 – 16 years | 44 | (3.0) | 54 | (5.3) | 2.16 | 1.43-3.25 | <0.001 | 1.77 | 1.15-2.73 | 0.010 | 1.24 | 0.75-2.07 | 0.402 |
| Sexual Abuse | |  |  |  |  |  |  |  |  |  |  |  |  |  |
|  | None | 1,376 | (92.6) | 912 | (89.2) | 1.00 | - | - | 1.00 | - | - | 1.00 | - | - |
|  | 0 – 11 years | 71 | (4.8) | 76 | (7.4) | 1.86 | 1.27-2.72 | 0.002 | 2.10 | 1.41-3.14 | <0.001 | 1.88 | 1.22-2.89 | 0.004 |
|  | 12 – 16 years | 39 | (2.6) | 34 | (3.3) | 1.46 | 0.89-2.40 | 0.131 | 1.76 | 1.06-2.92 | 0.029 | 1.45 | 0.82-2.57 | 0.197 |
| Bullying | |  |  |  |  |  |  |  |  |  |  |  |  |  |
|  | None | 1,045 | (71.8) | 594 | (59.3) | 1.00 | - | - | 1.00 | - | - | 1.00 | - | - |
|  | 0 – 11 years | 270 | (18.6) | 241 | (24.1) | 1.72 | 1.40-2.11 | <0.001 | 1.69 | 1.36-2.09 | <0.001 | 1.81 | 1.40-2.34 | <0.001 |
|  | 12 – 16 years | 140 | (9.6) | 166 | (16.6) | 2.28 | 1.77-2.94 | <0.001 | 2.14 | 1.64-2.78 | <0.001 | 2.25 | 1.66-3.04 | <0.001 |
| Any‡ | |  |  |  |  |  |  |  |  |  |  |  |  |  |
|  | None | 572 | (41.2) | 265 | (29.1) | 1.00 | - |  | 1.00 | - |  | 1.00 | - |  |
|  | 0 – 11 years | 649 | (46.8) | 526 | (57.8) | 1.77 | 1.46-2.14 | <0.001 | 1.79 | 1.46-2.19 | <0.001 | 1.86 | 1.48-2.34 | <0.001 |
|  | 12 – 16 years | 167 | (12.0) | 119 | (13.1) | 1.59 | 1.21-2.09 | 0.001 | 1.52 | 1.14-2.03 | 0.004 | 1.58 | 1.14-2.18 | 0.006 |

OR (1) Unadjusted

OR (2) Adjusted for age, sex, and ethnicity

OR (3) Adjusted for age, sex, ethnicity, IQ, parental history of psychosis, and parental social class at birth.

* n is number with complete data

‡ Any adversity, age of first occurrence
